# Innovative Strategies to Develop Chemical Categories Using a Combination of Structural and Toxicological Properties

**DOI:** 10.3389/fphar.2016.00321

**Published:** 2016-09-21

**Authors:** Monika Batke, Martin Gütlein, Falko Partosch, Ursula Gundert-Remy, Christoph Helma, Stefan Kramer, Andreas Maunz, Madeleine Seeland, Annette Bitsch

**Affiliations:** ^1^Department Chemikalienbeureilung, Dantenbanken und Expertensysteme, Fraunhofer Institut für Toxikologie und Experimentelle MedizinHannover, Germany; ^2^Institut für Informatik, Johannes Gutenberg-Universität MainzMainz, Germany; ^3^Institut für Arbeits-, Sozial- und Umweltmedizin, Universitätsmedizin GöttingenGöttingen, Germany; ^4^Institut für Klinische Pharmakologie und Toxikologie, Charité Universitätsmedizin BerlinBerlin, Germany; ^5^In Silico Toxicology GmbHBasel, Switzerland; ^6^Oncotest GmbHFreiburg, Germany; ^7^Institut für Informatik, Technische Universität MünchenMünchen, Germany

**Keywords:** non-animal methods, QSAR, read across, Predictive Clustering Tree (PCT) method, toxicological and structural similarity

## Abstract

Interest is increasing in the development of non-animal methods for toxicological evaluations. These methods are however, particularly challenging for complex toxicological endpoints such as repeated dose toxicity. European Legislation, e.g., the European Union's Cosmetic Directive and REACH, demands the use of alternative methods. Frameworks, such as the Read-across Assessment Framework or the Adverse Outcome Pathway Knowledge Base, support the development of these methods. The aim of the project presented in this publication was to develop substance categories for a read-across with complex endpoints of toxicity based on existing databases. The basic conceptual approach was to combine structural similarity with shared mechanisms of action. Substances with similar chemical structure and toxicological profile form candidate categories suitable for read-across. We combined two databases on repeated dose toxicity, RepDose database, and ELINCS database to form a common database for the identification of categories. The resulting database contained physicochemical, structural, and toxicological data, which were refined and curated for cluster analyses. We applied the Predictive Clustering Tree (PCT) approach for clustering chemicals based on structural and on toxicological information to detect groups of chemicals with similar toxic profiles and pathways/mechanisms of toxicity. As many of the experimental toxicity values were not available, this data was imputed by predicting them with a multi-label classification method, prior to clustering. The clustering results were evaluated by assessing chemical and toxicological similarities with the aim of identifying clusters with a concordance between structural information and toxicity profiles/mechanisms. From these chosen clusters, seven were selected for a quantitative read-across, based on a small ratio of NOAEL of the members with the highest and the lowest NOAEL in the cluster (< 5). We discuss the limitations of the approach. Based on this analysis we propose improvements for a follow-up approach, such as incorporation of metabolic information and more detailed mechanistic information. The software enables the user to allocate a substance in a cluster and to use this information for a possible read- across. The clustering tool is provided as a free web service, accessible at http://mlc-reach.informatik.uni-mainz.de.

## Introduction

At present, there is increasing interest in developing alternative methods for toxicological evaluations that do not require the testing of animals. In addition, particularly in Europe, several legislative imperatives drive an assessment of chemicals and products based on animal-free toxicological methods. For example, animal testing is banned in the cosmetic legislation and non-animal testing methods have to be used to fulfill the legal request for safe products (7th amendment to the European Union's Cosmetics Directive 76/768/EE).

Hence, both legislations underscore the need for non-animal tools and methods predicting the inherent toxic properties of chemical substances.

In REACH (Registration, Evaluation, Authorization, and Restriction of Chemicals), (Regulation (EC) No. 1907/2006), chemical risk assessment requires providing information on chemicals, the extent of which depends on the yearly production volume of the chemical. Whereas, the information required is clearly defined, the tools by which the information is gathered remain open and the legislative text [Annex XI of Regulation (EC) No. 1907/2006] stipulates only that animal experiments should be avoided whenever possible. Promising approaches, like grouping of substances and read-across (ECHA, 2014) are described under Section 1.5 of the above-mentioned Annex XI of Regulation (EC) No. 1907/2006. In this legislative text, similarity is defined based on a common functional group, common precursor or degradation product or a constant pattern in the potency of properties across the category.

Several guidance documents and their discussion (OECD, 2007a; ECHA, 2008, 2012, 2015; NAFTA TWG, 2012; Patlewicz, 2014) describe the principles but also the challenges of read-across approaches in detail. A quantitative read-across encompasses three steps (i) a definition on which similarity the search for analogs is based (physicochemical properties, chemical structure, shared mechanism, or a combination) (ii) a search for analogs with experimental data for the endpoint of interest (iii) a selection of the most similar substances out of step 1 and derivation of the missing point of departure for risk assessment.

In the context of defining a shared mechanism, the concept of adverse outcome pathways (AOPs)^1^ is becoming increasingly important (OECD, 2011). This concept has been developed as a structured approach to portray the linkage between initiating molecular events and the relevant adverse outcome at organism level (National Resarch Council/Committee on Toxicity Testing Assessment of Environmental Agents, 2007). AOPs assemble existing information into a concept, which includes knowledge on molecular interactions, cellular metabolism, and consequences of disturbances at check points.

Recently, after the Guidance Document on Developing and Assessing AOPs (OECD, 2013), OECD established an AOP knowledge base in September 2014. Furthermore, this approach has already been used to categorize nitrobenzene for their toxicity endpoint (Sakuratani et al., 2013b).

The combination of chemical structural similarity with shared mechanism of action was the basic conceptual approach of the project, which we will present in this publication. The aim of the project was to establish substance categories for complex endpoints of toxicity after repeated dosing. We explored several innovative strategies for setting up categories that would enable an estimate of repeated-dose toxicity supporting a read-across approach. Two databases were established containing physicochemical data including structure and molecular weight as well as toxicity data from repeated-dose testing. We adapted methods for clustering chemicals based on structural and on toxicological information by detecting groups of chemicals with similar toxic profiles and pathways/mechanisms of toxicity. We have evaluated the chemical clustering results, by assessing their chemical and toxicological similarities with the aim of identifying clusters with a concordance between structural information and toxicity profiles/mechanisms. The resulting clusters are discussed in detail in this publication and can be used for (quantitative) read-across. The overall tool is provided as a free web service^2^.

## Materials and methods

### Databases

Within this project the clustering is performed on a dataset consisting of data coming from a database on repeated dose testing of industrial chemicals (ELINCS) and the RepDose database^3^.

#### Data from ELINCS

The sources of the data are the regulatory documents from ELINCS (European List of Notified Chemical Substances), which is the new substance notification of the Chemicals Act (European Commission, 2009). The repository comprises new industrial chemicals registered in Europe between 1982 and 2008, which have been tested in subacute and/or subchronic studies. Practically, data on the substances were stored in the archive of the Federal Institute for Risk Assessment (BfR). The data comprise physicochemical and toxicological information. Access to the data from this source was restricted and regulated by a contract of confidentiality (Kalkhof et al., 2012).

This contract allowed the use of the data on the premise that the structures and chemical identity are held confidentially. The analysis of the confidential data was performed therefore only by the authorized authors. The data are of high quality because they have been obtained under defined internationally accepted experimental OECD-standards (OECD, 1998a,b and former versions). In addition, as a rule, regulatory scientists of an EU member state reviewed the studies and their results and assigned reliabilities. The full ELINCS repository is available to all European Competent Authorities for chemical assessment.

To enable the analysis of the ELINCS data, the studies were stored in a database format in accordance to the RepDose database. Only studies performed with chemical substances with a purity ≥90% were entered in this final database. The purity of 90% was also applied as prerequisite for further study selection in this analysis. Studies with dermal or inhalation route of exposure were not available in the digital version of ELINCS data.

Overall, the full database includes 540 substance entries. All studies are compliant with the OECD guidelines 407 and 408 (OECD, 1998a,b and former versions). ELINCS tabulates substances by the registration number with the standard format: xx-xx-xxxx. The first digits represent the year of notification, followed by two digits representing the country of notification. The last four digits allow sequential numbering of individual dossiers (Barabair et al., 2009).

#### Data from the RepDose database

The RepDose database is a relational database on toxicological animal testing after repeated administration. Information from publically available peer-reviewed reports and original publications on existing organic chemicals with defined structures (no polymers, no mixtures) is collected in the RepDose database. Chemicals were selected if evaluations exist e.g., by German MAK committee, in EU Risk Assessments on existing chemicals, in OECD Existing Chemicals Screening Information Data Sets (SIDS), or in the eChem-Portal (REACH). In addition, projects like the development and evaluation of TTC concepts with a special focus on inhalation application contributed information to the database. Originally, the database was funded by a CEFIC LRI project with the basic idea of providing a user-friendly tool for setting up structure-activity relationships as well as other supporting methods for a simplified and scientifically sound risk assessment (Bitsch et al., 2006). The data within the RepDose database are organized by information types: physicochemical properties of the test chemical, study design including guideline compliance, purity and scope of examination, observed effects at the related doses [effect Lowest Observed Effect Level (LOELs)] based on glossaries for organs and related effects. Overall, the RepDose database contains about 850 chemicals with 2900 related studies of which about 400 studies were selected as being guideline-conform to oral or inhalation subacute or subchronic studies with rats. The guideline compliance is coded in the RepDose database analog to Klimisch Codes with *A*—*guideline conform*—and *B*—*minor deviations from guidelines*—being considered for this project.

### Dataset

As mentioned above, the following selection criteria were applied to compile a consistent dataset out of the two sources:

– duration is subchronic (84–99 days) or subacute (28–32 days), route of application is oral or inhalation.
– studied species is rat.
– studies are highly reliable as conducted in conformity to (current) guidelines.
– purity of the substances is at least 90%.

The organization of toxicological data in both databases follows the same basic structure; the data are organized by an organ toxicity split into subgroups according to similarities at the phenotypic and the mechanistic level. All target organs and effects are named according to a thorough chosen and curated glossary.

The final dataset comprises 1022 studies (64 both, 557 subacute, 278 subchronic) for 899 organic industrial chemicals. The toxicological information of a chemical is taken from all studies available.

#### Curation and refinement of the dataset

We developed a common glossary for all endpoints, which were applied to the toxicological findings.

In developing the common glossary, it became obvious that there was a conflict between the level of granularity and the density of data in the matrix and a compromise had to be found. The following procedure was followed:

At the beginning, LOELs for up to 460 different organ-effect-combinations were extracted from the databases. The results were characterized by a very sparse matrix with many missing LOELs, where there were different reasons for missing LOELs such as no finding, not investigated and no information available. To reduce the number of missing values we introduced first a cut-off of 5% only organ effect combinations that occur at least for ≥5% of all substances that were included in the dataset. In a second, related step, organ-effect combinations were merged according to their toxicological relationship and based on toxicological expert knowledge. For illustration liver-degeneration, liver-hypertrophy, liver-inflammation-regeneration, and remaining liver effects were aggregated to liver effects and effects on erythrocytes, hematocrit, and hemoglobin were collected as rbc effects. Thereby, a plausible compromise between specific but not too sparse information was obtained. In the final dataset, every substance is characterized by 28 endpoint/organ combinations.

In case of multiple LOELs for one study due to different organs affected the lowest LOEL was taken and was declared the study LOEL.

#### Discretization of data

In addition to the toxicological profile, we decided to include the toxicological potency using LOEL values. In a further step, these LOEL values were categorized into high-potency and low-potency as described in this section.

**Figure 1:**
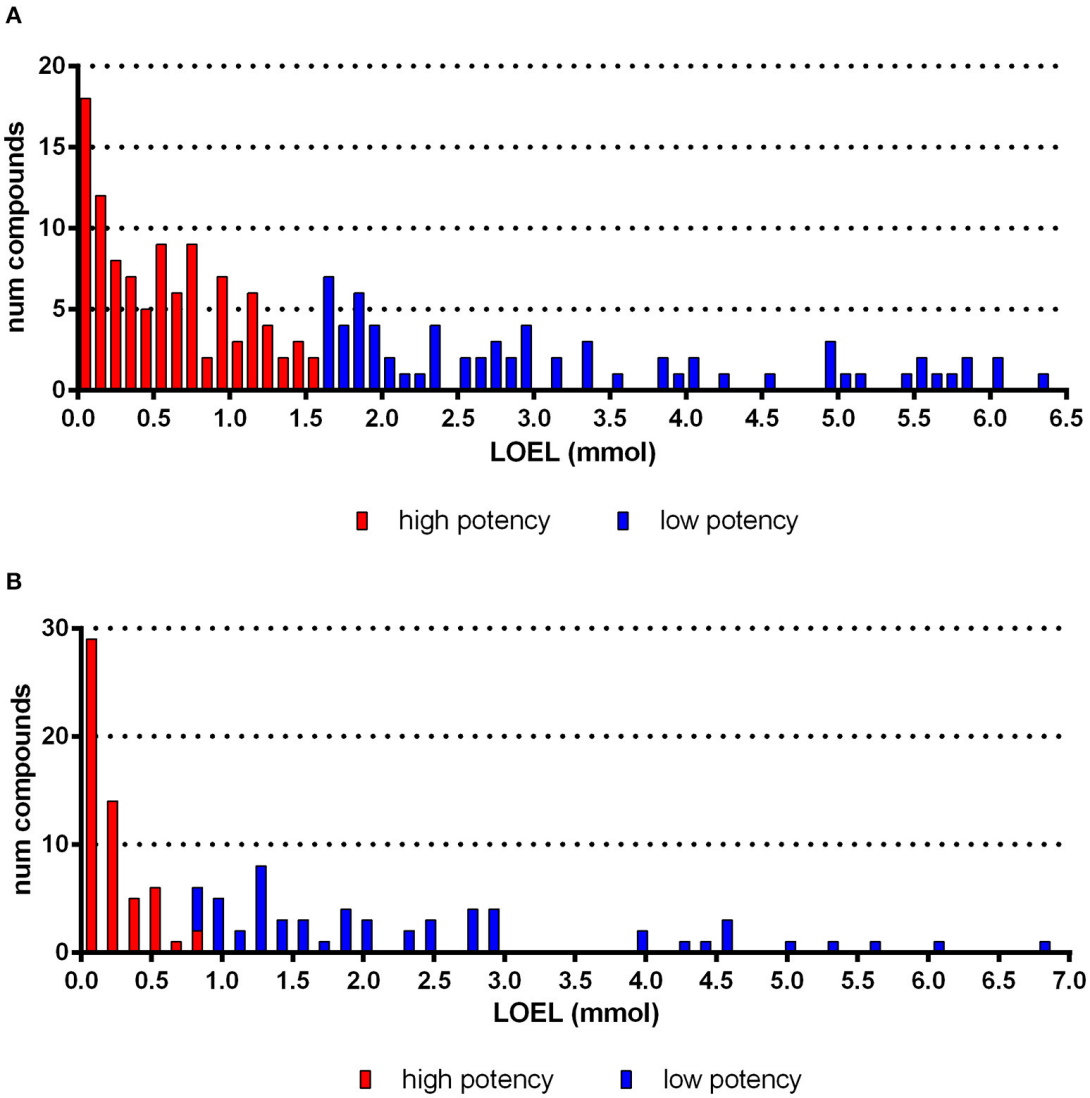
**Histogram of compounds according to subacute (A) and subchronic (B) LOEL values for the endpoint “red blood cells.”** For this example, the discretization approach yielded a threshold of 1.57 mmol **(A)** and 0.78 mmol (**B**, half of the subacute threshold).

One of the challenges of modeling *in vivo* data is the high uncertainty of experimentally derived endpoint values. Moreover, aggregating the dataset from numerous studies introduces more noise. Hence, to simplify modeling, we converted the numeric data (LOELs) to binary nominal data with class values for high-potency and for low-potency for each endpoint (organ-effect combination). As toxicological effects are related to the number of moles present at the site of actions, the doses were converted to moles of chemicals/kg bw/day taking into consideration the molecular weight of the chemicals. We developed a clustering-based discretization method that automatically detects a threshold specifically for each endpoint: Compounds with a LOEL lower or equal to this threshold are categorized as high-potency compounds; compounds above this threshold are categorized as low-potency compounds. An example is given for red blood cells in Figures 1A,B. The main idea of our approach is to adjust the threshold to the existing data distribution.

Our technique produces a balanced ratio of high-potency and low-potency class values, which is often preferable for modeling (Japkowicz and Stephen, 2002). Therefore, we manually limit the threshold to a fixed range of 1.5–2.0 μmol (for subacute studies). Subsequently, our clustering method determines a threshold dynamically within this range, in contrast to the rigid threshold that is applied by, e.g., Equal Frequency Discretization (Dougherty et al., 1995). This method yields a mean ratio of 49% high-potency compounds in the overall dataset. The distributions of LOELs for effects on red blood cells are shown as example in Figures 1A,B.

The dataset used in this publication is composed of subacute studies with study durations of 28–32 days and subchronic studies with 84–99 days. Overall the distribution of our data supports the assessment factors proposed by ECHA (2012) showing a factor two between subchronic and subacute effects. The analysis of effects on red blood cell is given as example (Figures 1A,B). Hence, in the further processing of the data we have adjusted the threshold for subchronic studies according to ECHA guidelines to take the increased study duration into account (ECHA, 2012).

#### Handling of missing values

**Figure 2:**
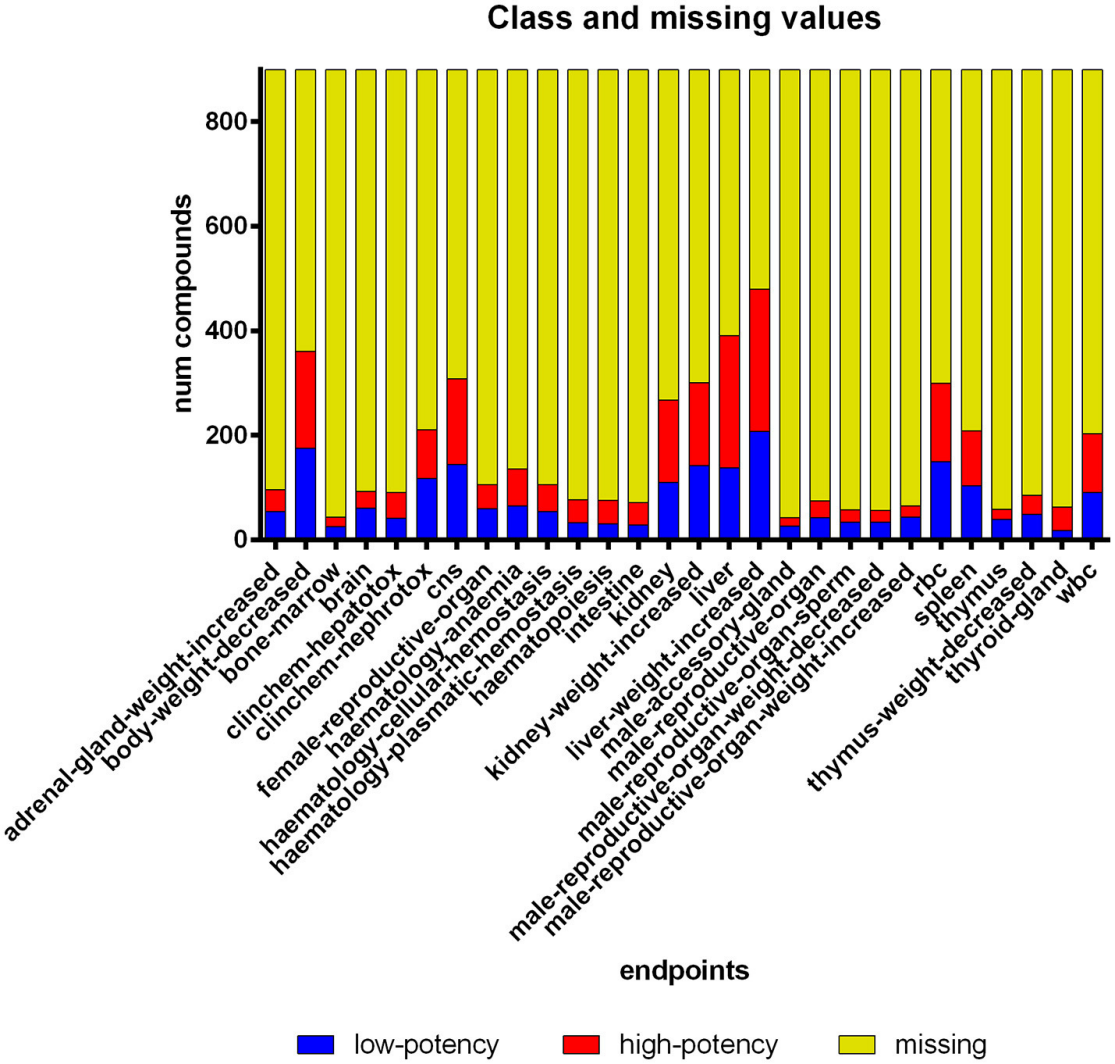
**Compound histogram for each endpoint in the dataset**. Eighty-two percent of the LOEL values are missing. The discretization approach produced a balanced class distribution (49% high-potency compounds).

As described above, the dataset has been compiled from various studies for a multitude of chemicals. This implies that not all endpoints were affected and/or tested for every chemical. In total, 82% of the compound endpoint pairs are “missing,” which means that information on these endpoints was not available (Figure 2). To make the most of the available information and to enable clustering in the first place, we are using a method for so-called imputation (Schafer, 1997), i.e., a method substituting missing values by “best guesses.” The imputed values are chosen, taking advantage of known variable correlations. One approach for imputation is employing machine learning models, i.e., learning a classifier to predict the missing values, which has been shown to yield good results (e.g., Jerez et al., 2010). As our dataset has multiple nominal endpoint values for each compound, a Multi-Label-Classification (MLC) algorithm is required that predicts multiple endpoint values simultaneously (Tsoumakas and Katakis, 2007). We have selected Ensemble of Classifier Chains (Read et al., 2011) as MLC model to predict the missing values in our dataset. The imputed values are exclusively used as input for the clustering algorithm (i.e., the PCT algorithm is applied to a “filled up” version of the dataset without missing values). The analysis of the resulting clusters is restricted to the non-imputed data including missing values.

#### Features

Features were selected according to their toxicological relevance in an iterative process during cluster evaluation. The features employed by the PCT serve as description for the resulting clusters. Hence, we chose a combination of structural features with the two intuitive physicochemical (PC) descriptors molecular weight and log P (computed with Open Babel O'Boyle et al., 2011). A range of additional PC descriptors were tested, but they were finally deselected by expert judgment to retain interpretability. We decided to use lists of pre-defined structural features instead of computing all or a subset of relevant substructures in a given dataset of structures. The predefined structural feature lists include functional groups and other known structural alerts and have further the advantage that a short description is provided for each structural feature. We have composed three lists that are included in Open Babel to create fingerprints. The lists include 166 (MACCS keys; Durant et al., 2002), 55 (FP3; Haider, 2010), and 307 (FP4) structural features each. The fact that some structural features occur in more than one list does not affect the building of the PCT model.

### Clustering

On the basis of this final dataset, we determine chemical categories by applying the method of Predictive Clustering Trees (PCTs; Blockeel and De Raedt, 1998). This method splits up the dataset into clusters of similar compounds, while at the same time it can also provide a prediction model that assigns untested compounds to the detected clusters.

PCTs are part of a general framework, called predictive clustering, that unifies clustering and prediction. As in clustering, predictive clustering seeks clusters of examples that are similar to each other and dissimilar to the examples in other clusters. In addition, a predictive model is associated to each cluster, which can be used to assign new compounds to clusters and to provide predictions for them. PCTs can be considered as a generalization of standard decision trees and yield a hierarchical clustering tree, each tree node corresponding to a cluster. The root node of the tree corresponds to a cluster that contains the entire dataset, and is recursively partitioned into smaller clusters while moving down the tree. The leaves represent the clusters at the lowest level of the hierarchy and each leaf is labeled with its cluster's prediction. In each node, a test is applied that divides the compounds of the current cluster into two sub-clusters. For example, it could be tested whether a chlorine atom occurs in the chemical. Thus, a test in a node not only represents a decision criterion, but also a description of the sub-clusters formed in this node. PCTs are induced by a standard procedure for the top-down induction of decision trees (Breiman et al., 1984).

The PCT approach takes as input a set of instances consisting of (i) the descriptive attributes, which are to be used in the cluster description, i.e., the tests that appear in the PCTs' node and (ii) the target attributes which are to be predicted from the descriptive attributes. In our case, the instances represent the chemicals, the descriptive attributes denote structural properties and the target attributes the discretized toxicological properties (low or high potency).

The approach, which included several iterations, can be described as follows. The main loop searches for the best acceptable test, i.e., the best structural attribute value that can be put in a node of the tree. To select the best test, the method scores the tests according to the reduction in variance they induce on the instances associated to the node. PCTs compute cluster variance as the sum of the squared pairwise distances between the toxicological values of (sub-)clusters. At each node of the tree, the test that maximizes the variance reduction is selected. This is expected to maximize cluster homogeneity with respect to the target attributes and improve the predictive performance of the tree. If the best test is acceptable with respect to a stopping criterion, the algorithm creates a new internal node and calls itself recursively to construct a sub-tree for each cluster in the partition induced by the instances. If no acceptable test can be found, the algorithm creates a leaf. The stopping criterion used in this work is inspired by that of standard decision tree learners: the minimal number of compounds in a leaf is set to three, and a statistical *F*-test checks whether the reduction of variance of the toxicity values obtained with a split is significant at a (quite tolerant) significance level of 0.125. Both parameter values have been set by expert judgment. To produce a PCT model with a single clustering tree, we have disabled ensemble settings that would create multiple trees (bagging or random forests). Moreover, we have prevented the algorithm from truncating the tree after model building by disabling post-pruning.

**Figure 3:**
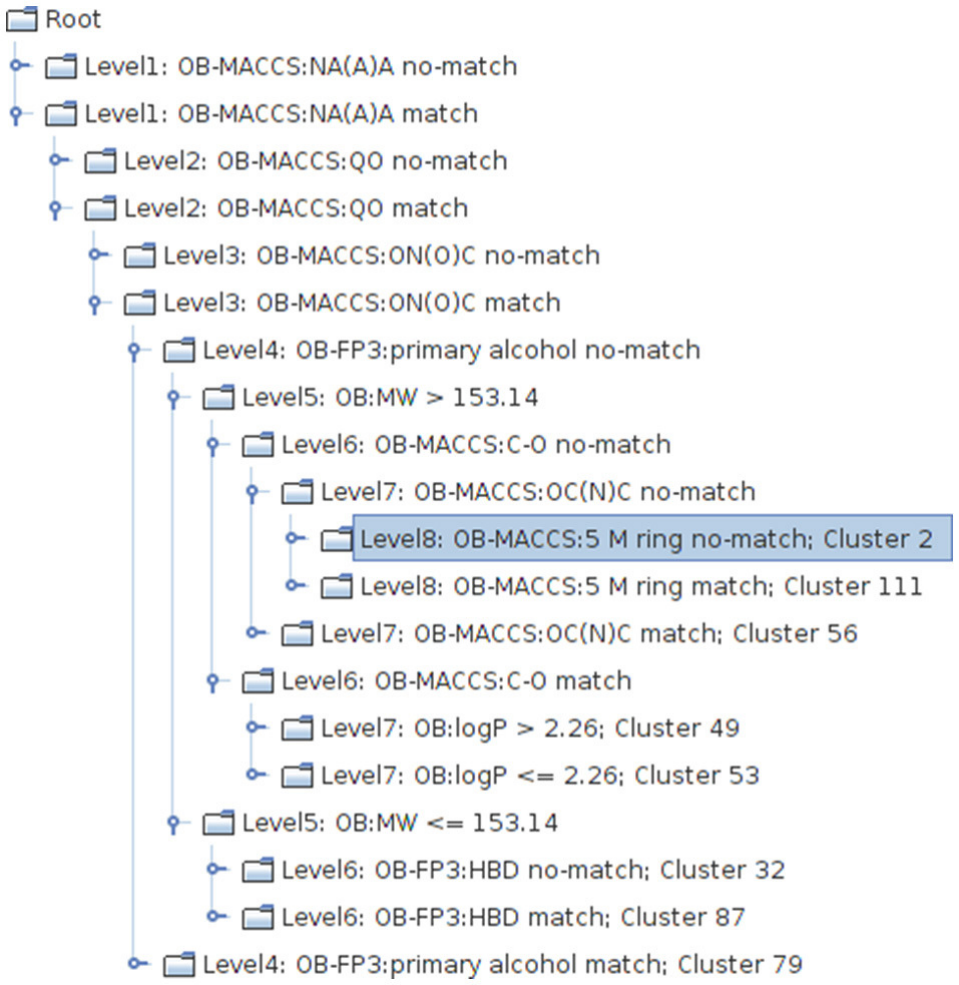
**Excerpt from the PCT clustering result, visualized with the tree view implemented in the CheS-Mapper tool (Gütlein et al., 2012)**. One structural feature is used at each level of the tree to divide chemicals into sub-groups with high toxicological similarity.

The resulting PCT represents a clustering that is homogenous with respect to the target attributes and the nodes of the tree provide a symbolic description of the clusters (Figure 3). The model can be applied to unseen compounds on a freely available web page^4^.

### Visual presentation and analytics

**Figure 4:**
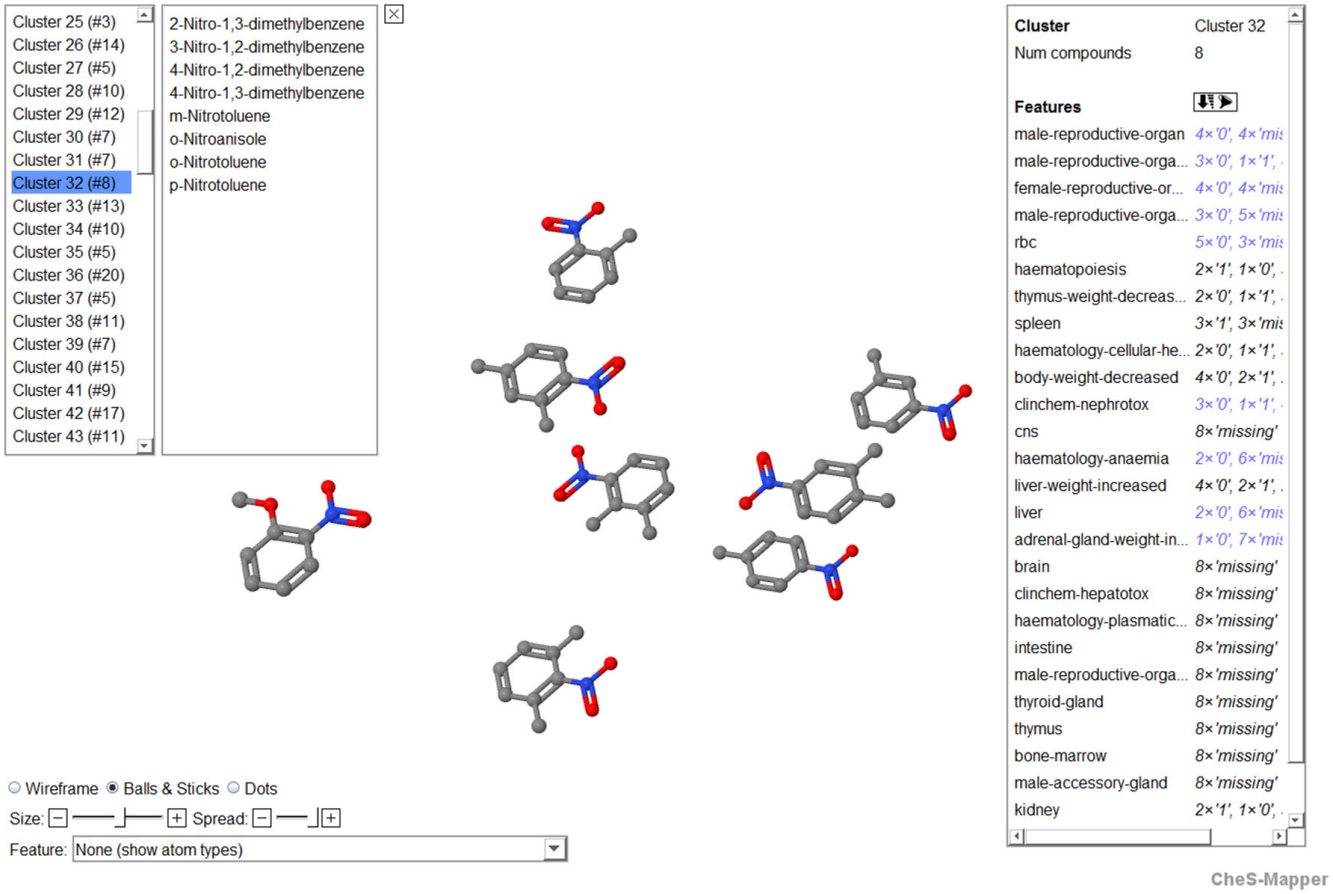
**Screenshot of CheS-Mapper showing cluster 32 that includes eight compounds (nitrotoluenes and dimethylnitrobenzenes)**. In the upper left corner, all clusters are listed and the members of the selected cluster 32 are shown. On the right side of the application screen, the user can select features for which the frequencies are depicted, in this example the frequencies of the discretized organ-effect combinations (0, low potency; 1, high potency, missing) are displayed. In the lower left corner of the screen, features are given to change and adopt the molecular visualization.

The 3D viewer CheS-Mapper (Gütlein et al., 2012) was applied and extended for inspection of the clustering results (Figure 4). It is a freely available application that embeds a dataset of chemical compounds into 3D space, so that compounds with similar feature values are located close to each other (http://ches-mapper.org). CheS-Mapper is normally used to calculate its own features and clusters. However, in this case, we explored the dataset with precomputed features and cluster assignments. The tool was employed to inspect the structural similarity of cluster compounds. Moreover, dedicated highlighting and filtering functions allow analyzing how the rat toxicity values are distributed within the dataset and/or single clusters.

We have further extended CheS-Mapper with a plugin that supports hierarchically clustered data. An interactive tree view (Figure 3) is provided alongside the 3D viewer. The selection of the current compounds or clusters is dynamically synchronized in both views.

### Calculation of the “toxic value”

In order to obtain a unidimensional characterization of the substance toxicity, we aimed at condensing all the information on a substance to derive a single value. To this end, grades were assigned to each of the toxicological endpoints, whereby 0 characterized a, for whatever reason, missing toxicity, 1 a low potency of toxicity, and 2 a high potency of toxicity (as described above). By adding up the grades we obtained a single value named “toxic value.” At the end, every substance had its “toxic value.” We used this value to investigate the relationship between molecular weight, log P and the toxicity.

### Assessment of clustering results with respect to toxicological plausibility

The assessment of the clustering results has been done in a two-dimensional approach. First, the homogeneity of the chemical structures was considered, using chemical expertise. The structural similarity in a cluster can be expressed in percent of chemicals having a common structural feature and the toxicological similarity can be assessed based on the number of chemicals in the cluster with one or more common toxicology endpoints or target organs for toxicity. The similarity in structure compared to the lead structure in the cluster, was expressed as a percentage. Secondly, we looked at the toxicological similarity, driven by toxicological expertise. The similarity in toxicity compared to the lead toxicity in the cluster was expressed as a percentage. We categorized the clusters in the following way. Clusters were Category 1 when a common endpoint (100% toxicological similarity) and well-defined related structural features (100% structural similarity) were present including knowledge about the mode of action. Category 2 are clusters with well-defined related structural features (100% structural similarity) and a common toxicological profile (of 75 and up to 99%) and Category 3 are clusters with well-defined related structural features (100% structural similarity) and a less well expressed common toxicological profile (up to 74%). In addition, an assessment of substances with high toxicological similarity but low structural similarity has been performed.

## Results

### General description of the dataset

The database contains 899 chemicals with a wide range of physicochemical properties and a broad spectrum of toxicological endpoints. The molecular weight of substances ranges from 32 to 1297 g/mol with a median value of 215 (25th percentile of 147; 75th percentile 315); the log P from −17.4 to 12.8 with a median value of 2.4 (25th percentile 1.5; 75th percentile 3.9).

In general, the main target organs for toxicity are liver (with toxicological results in 51% of the chemicals), kidney (14%), and CNS (11%). The toxicological profile of single clusters is defined by a combination of organs and endpoints representing the characteristic toxicological fingerprint of the cluster. The analysis of the most common target organs per cluster revealed that over 50% of the clusters have liver as major common target organ. Thus, most of the chemicals in these clusters have effects on liver histopathology, liver weight and/or clinical chemistry related to liver toxicity. The common toxicological fingerprint might be more specific due to additional specific effects.

The toxic values ranged from zero to 32 points. Among the substances in the upper quartile of toxic values are 9,10-anthraquinone, acrolein, p-chlorobenzotrichloride, N,N′-diphenylguanidine, 2-mercaptobenzimidazole, trinitrofluorenone, tetrahydro-2-furanmethanol and 2,2′-dimethyl-4,4′-methylenebis(cyclohexylamine). These substances are obviously not structurally related.

### Relationship between physicochemical data and toxicity

**Figure 5:**
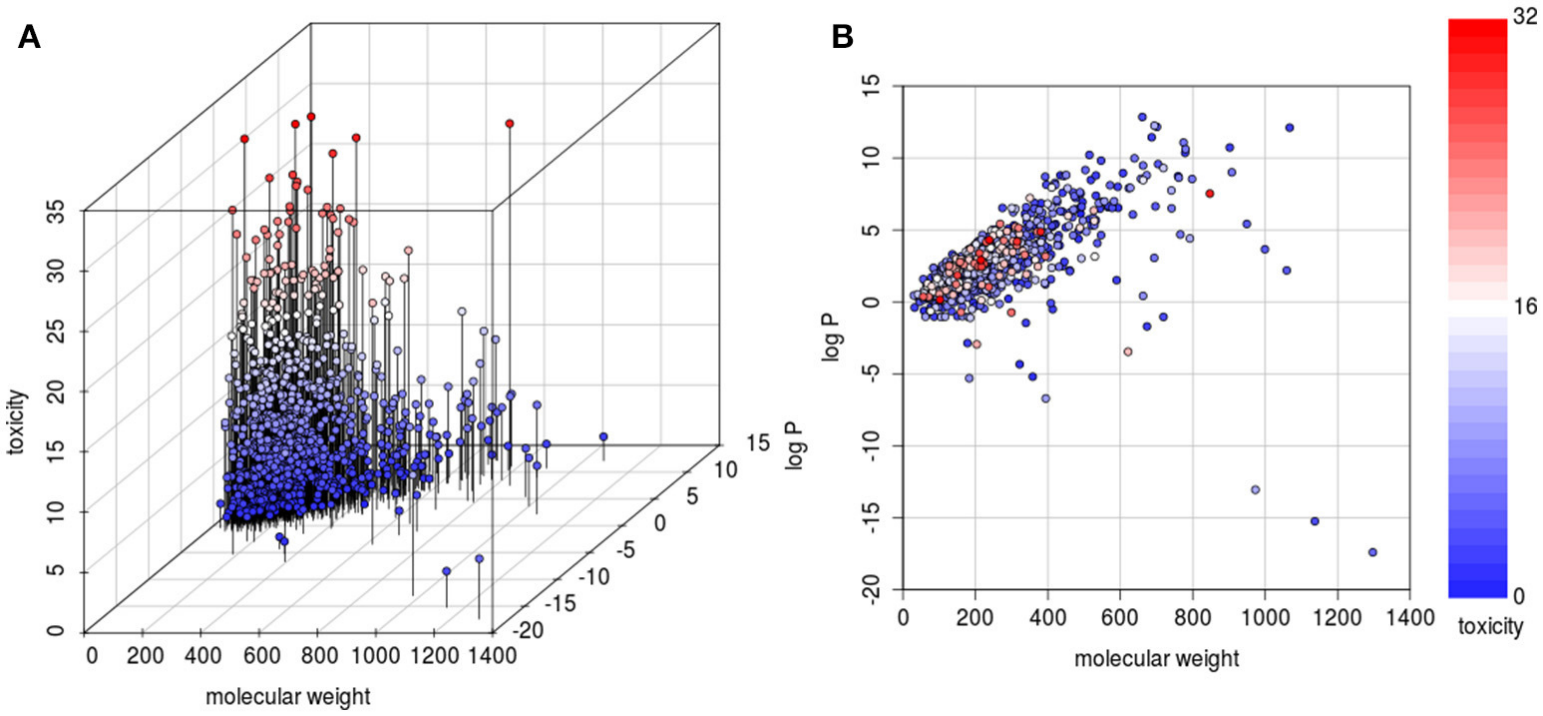
**3D (A) and 2D (B) scatterplots to visualize the relationship between physicochemical properties (molecular weight and log P) and toxicity**. The 3D plot has an additional axis to separate compounds with identical physicochemical values according to their toxicity. There are few highly toxic compounds with high molecular weight. There is no monotonic correlation between log P and toxicity, but the scatterplot shows that highly hydrophilic substances (log P below 0) and highly lipophilic substances (log P above 5) exhibit a low toxicity.

In evaluating the impact of physicochemical data on toxicity, we analyzed the relationship between molecular weight, log P and toxic values. As it can be seen from the three-dimensional graphical analysis in Figure 5, only few substances with high molecular weight (above 500 g/mol) had a toxic value above the mean (< 5%). All highly toxic substances (defined as a toxic value above the upper quartile of 24 points), with only one exception, have a molecular weight below 400 g/mol. Thus, molecular weight is a predictor to discriminate between toxic and less toxic substances. When analyzing the log P it evolved that it has a lower discriminatory power. However, it can be said that in this database highly toxic substances have a log P between 0 and 5. The log P values were estimated as measured values (same validated method resulting in the same systematic error) were not available for all substances. Thus, for the correlation analysis it was judged more acceptable to use estimated values.

### Clustering results and toxicological similarity

Clustering assigned the 899 chemicals to 119 clusters. The mean cluster size is 6 chemicals per cluster, ranging from 3 to 24 chemicals per cluster. To assess the outcome of the clustering, the members of a cluster were categorized according to their chemical structural similarity and toxicological similarity.

From the 119 clusters, 29 clusters (24.4%) contain chemicals with a structural similarity of 100%. Among these, chemicals of 8 clusters have a toxicological similarity of 100% (category 1), in an additional 9 clusters the toxicological similarity is 75% and above (category 2), and finally 12 clusters have a less well-expressed common toxicological profile (up to 74%, category 3; Supplementary Table 1). Thus, overall, the clustering process resulted in toxicological meaningful clusters in 60% of the clusters with a structural similarity of the cluster members of 100%.

#### Selection of clusters for qualitative and for quantitative read-across

Among the clusters with 100% structural similarity, 8 clusters are assessed as having 100% toxicological similarity (category 1). These 8 clusters of category 1 can be seen as candidates for a qualitative read-across. Furthermore, in addition, 9 clusters of category 2 and further 11 clusters of category 3 (100% structural similarity/less than 75% toxicological similarity) were evaluated with respect to the range of LOELs observed in the cluster.

When analyzing the LOELs, the ratio between the upper and the lower end of the range varied between 1.2 and 63,875, indicating that a quantitative read-across is not justified even for each cluster of category 1. On the other hand, there are clusters of category 2 and even category 3 (chemical similarity/ toxicological similarity below 75%) with a ratio of below 15 between the upper and the lower end of the range (Supplementary Table 1). In Table 1 we have listed the clusters with a low ratio (< 15) between the upper and the lower end of the range of LOELs which we would like to propose as candidates for a quantitative read-across.

**Table 1:** **Proposed clusters for quantitative read-across with a ratio of geometric mean/lower end of the range ≤ 5**.

**Number of cluster**	**Structural feature**	**Common target**	**LOEL geometric mean [mmol]**	**LOEL range [mmol]**	**Ratio (geometric mean/lower end of the range)**
61	Azoles	Kidney	0.52	0.49–0.59	1.1
13	Methylphenols	CNS	0.58	0.32–1.39	1.8
107	Alcohols	kidney weight, liver weight	0.03	0.01–0.09	3
53	Nitroaromates	CNS	0.77	0.24–2.01	3.2
16	Aromatic phenols without other substituents	Liver	0.87	0.23–13.50	3.8
76	Nitrophenols and -anilines	Liver	0.21	0.05–0.66	4.2
44	Carboxylether	Liver	1	0.22–3.85	4.5
75	Glycolether	Liver, kidney	0.75	0.14–2.84	5

So far, the clustering results were evaluated on the general cluster descriptions (similarity and quality) and their distribution. However, it should be considered that substances with one common structural feature (e.g., reactive group) but different additional structural features (e.g., hydrophobic/hydrophilic side chains) could exhibit different toxic effects. Therefore, in an additional analysis, it was evaluated if and how the clustering algorithm separated such compounds according to their differences in toxicity. We performed this analysis for two substance groups, nitro compounds and alcohol dehydrogenase substrates such as ethylene glycols and alcohols.

### Clusters identified for specific structural features

#### Nitro-group containing compounds

**Figure 6:**
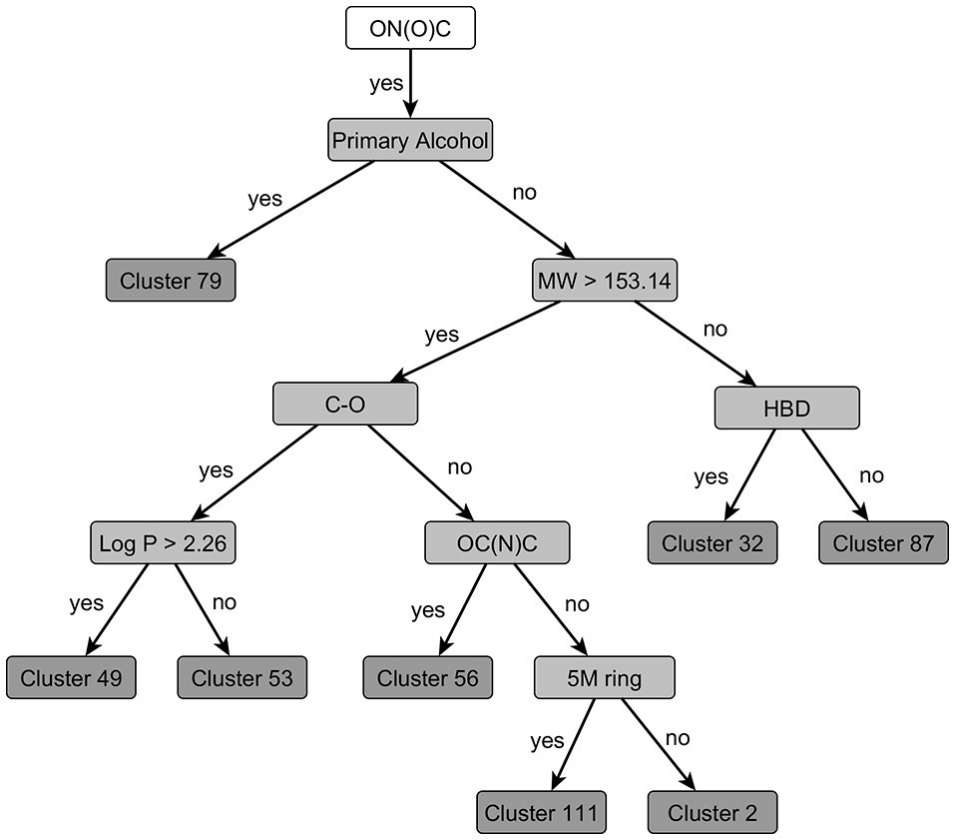
**Decisions to be taken for clustering of nitro-group containing compounds [defined by the SMART-Code ON(O)C]**. At each node the feature (structural feature, molecular weight, or log P) with the highest discriminative power is selected and the subsequent clusters are formed if their toxicity profiles are significantly different (*p* = 0.125). If no selective feature resulting in a significantly different toxic cluster can be identified, the clustering terminates by a final cluster, e.g., Cluster 49. The following structural features as defined in the OB FP4 list (O'Boyle et al., 2011) are used in this clustering tree: HBD, hydrogen bond donors; OC(N)C, Aliphatic O joined by any bond to C with joined with N and C; C-O, C-O single bond; 5M ring, any five-membered rings (the explanations for the SMARTS codes of the branches can be taken from the Supplementary Material).

Within the dataset, 64 compounds contain a nitro-group. Two structures are aliphatic nitro-compounds clustering separately and are not discussed in the following. Further three structures contain a nitro-group bound to other heteroatoms and also cluster separately (Cluster 42). The remaining 59 structures cluster according to the clustering tree depicted in Figure 6.

The clustering tree assigns the 59 structures into eight clusters based on their structural properties and their differences in toxic effects. The PCT algorithm selects a discriminant structural feature at each possible node of the tree, but separates the substances only into two groups if their toxicity profile is significantly different. Otherwise the possible node will form a leaf of the tree, in other words, it becomes a final cluster.

The resulting nitro-group containing clusters are characterized for their differences in structure and toxicity in Table 2. In addition to the automated clustering, cluster 32 can be split into more toxic nitrotoluenes and less toxic dimethylnitrobenzenes (Figure 6). Overall, two relatively toxic clusters with LOELs below 0.1 mmol/kg bw/d are identified. Another four clusters with LOELs about 0.1–1 mmol/kg bw/d have a medium toxicity and further 3 clusters with LOELs above 1 mmol/kg bw/d can be regarded as having a low toxicity. The toxicological profiles of these clusters differ not only in potency but also for the affected targets, e.g., nitroanilines show a high toxicity to male reproductive organs, spleen and hematopoiesis, whereas nitroaromatics with a 5 molecular ring in addition target liver, hematopoiesis and induce anemia. Nitroaromatics with heterogenic side chains as collected in Cluster 49 do not induce anemia but show some mixed toxicity in male reproductive organs and spleen. From a toxicological point of view, the resulting 9 clusters can be divided into 5 clusters with specific toxicity to male reproductive organs, spleen, liver, and/or hematopoiesis/anemia and other 4 clusters exhibiting a mixed and unspecific toxicity.

**Table 2:** **Characterization of clusters containing nitro compounds: differences in structure and toxicity profile (major targets and geometric mean LOEL values)**.

**Cluster number**	**Number of chemicals**	**Structure**	**Mean molecular weight of the cluster**	**Major targets**					**LOEL [mmol] (geometric mean; range)**
				**Male reproductive organs**	**Spleen**	**Liver**	**Hematopoiesis/ anemia**	**Mixed toxicity**	
2	16	Nitroanilines, nitrobenzenes	217 ± 82	+	+		+		0.05 (0.0005–2.00)
111	5	Nitroaromatics (containing 5M-ring)	302 ± 139			+	+		0.06 (0.003–0.39)
49	15	Nitroaromatics (heterogenic side chains)	330 ± 139	+	+			+	0.21 (0.002–2.94)
32a	4	Nitrotoluenes	141	+					0.26 (0.10–0.40)
87	3	Nitrophenol, nitromethylanilin, nitrobenzeneamine	143 ± 8					+	0.40 (0.10–1.30)
56	4	Heterogenic structures	224 ± 22					+	0.41 (0.07–0.82)
79	5	Nitroaromatics (at least two side chains)	238 ± 35	+			+	+	1.13 (0.50–2.80)
32b	4	Dimethylnitrobenzenes	151					+	1.61 (1.00–2.00)
53	3	Nitroaromatics	227 ± 38					+	2.35 (1.20–4.00)

+ denotes the presence of findings for this target.

**Figure 7:**
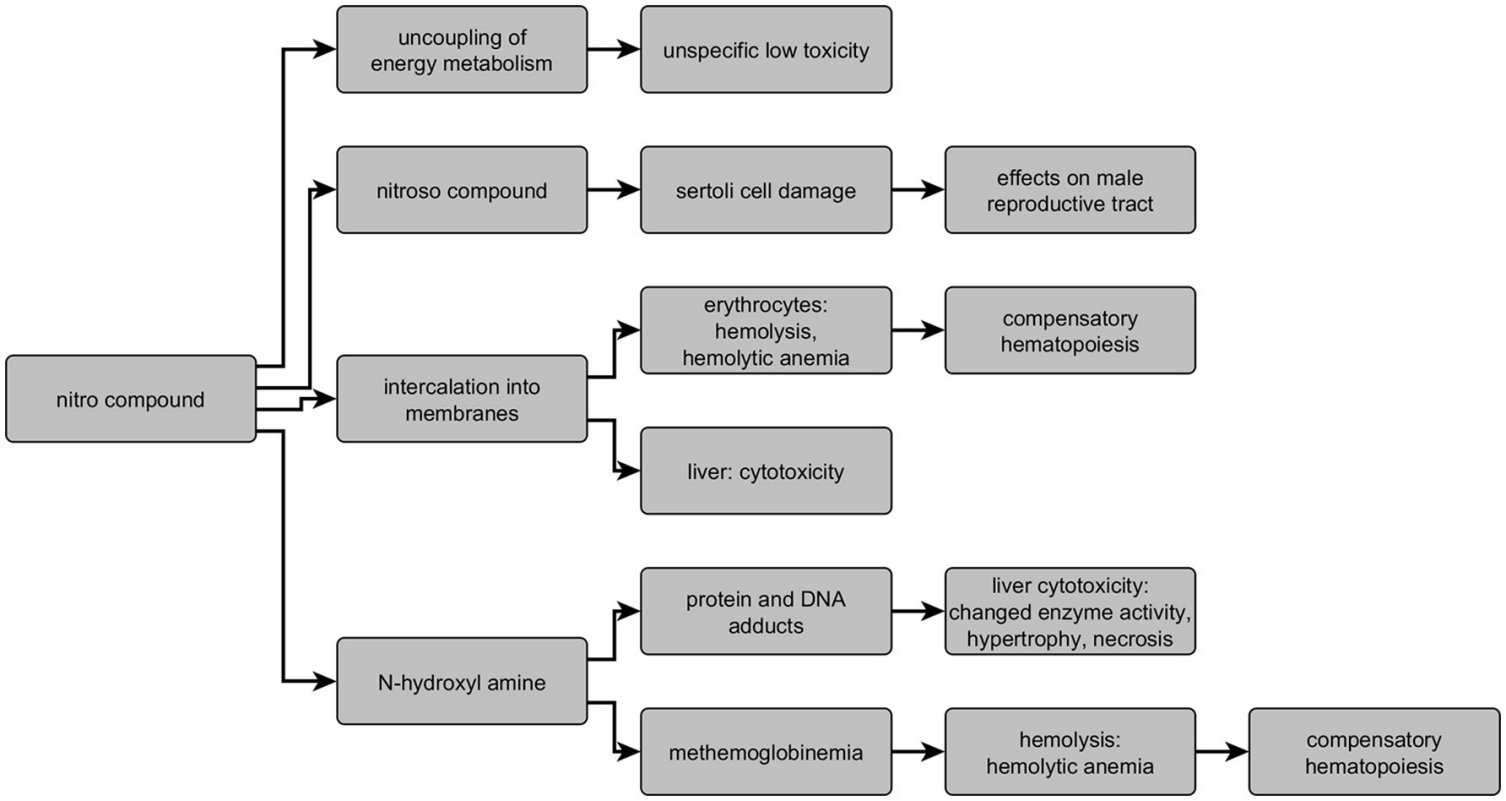
**Overview on modes of action and targets of nitro compounds indicating key metabolites and effects**.

In the literature for nitro-containing compounds, several targets/modes of action are described: erythrocytes, testis, liver, and oxidative phosphorylation. The HESS system provides an overview on the different modes of action and describes structural boundaries based on active chemicals collected in the respective database. Overall, five different adverse outcome pathways to predict the mode of action of nitrobenzenes are contained in the HESS system (Sakuratani et al., 2013a,b): hemolytic anemia with and without methemoglobinemia, hepatotoxicity based on two different mechanisms, and testicular toxicity, as well as the model on energy metabolism dysfunction of nitrophenols/halophenols. An overview on the different modes of action/targets and the key metabolites as well as targets is given in Figure 7. Toxicity based on N-hydroxylamine formation is observed with compounds clustered in Cluster 2 resulting in a relative high toxicity (liver and hematology). Cluster 111 comprises highly toxic compounds as well and is characterized by liver toxicity and hemolysis. This is not based on the formation of methemoglobin, but intercalation of the nitro compounds itself (Sheetz and Singer, 1976). The AOP resulting in testicular toxicity based on nitroso metabolites is well described in literature, but only represented by few chemicals in the HESS system. Within the current dataset, it can be observed with substances clustered in cluster 2, and 32a, supporting the relevance of this mechanism in addition to the few positive compounds described in the HESS system. Rather unspecific target organ toxicity results from compounds acting as uncouplers of energy metabolism. The chemicals clustered within cluster 49, 53, 56, and 32b, showing unspecific medium to low toxicity, act via this mechanism. Additionally these clusters comprise also other substances like the antibiotics nitrofurazone and nitrofurantoin, also not exhibiting specific target organ toxicity. Overall, the combination of structural and toxicological fingerprints yields well described and distinguished groups of nitroaromatic compounds following different types of AOPs.

Within this in-depth analysis of the aromatic nitro compounds, it became however evident that the clustering strongly relies on the underlying data: if discriminant structural features are not available within the used features or if the toxicological data is not specific enough, then clusters cannot be identified. For an optimal clustering result, optimal data are thus indispensable.

#### Ethylene glycols and alcohols: a mixed cluster

The Cluster 11 consists of 24 members being ethylene glycols and alcohols (Table 3). From a chemical point of view, ethylene glycols, and alcohols are different, however, the members have a common biological feature: they are substrates for alcohol dehydrogenase (ECETOC, 1995) and share toxicological endpoints such as kidney, liver, red blood cells, and spleen indicating a structural grouping with toxicologically meaningful results.

**Table 3:** **Characterization of substances in Cluster 11: Chemicals and their toxicity profile based on major targets and LOEL (low observed effect level)**.

**Cluster number**	**Chemical name**	**Molecular weight**	**Major targets**				**LOEL [mmol] geometic mean**
			**Kidney**	**Liver**	**RBC**	**Spleen**	
11	Ethylene glycol	62.07	+	+			4.04
	Diethylene glycol	106.12	+	+			2.83
	Diethylene glycol monoethyl ether	134.17	+	+	+	+	29.21
	Ethylene glycol monomethyl ether	76.09	+			+	0.92
	Ethylene glycol monoethyl ether	90.12	+	+	+	+	2.27
	Ethylene glycol monoisopropyl ether	104.15			+	+	0.56
	Triethylene glycol	150.17	+		+		10.14
	Triethylene glycol monomethyl ether	164.2		+			2.44
	Propylene glycol	76.09					2.99
	2-propylene glycol-1-methyl ether	90.12		+			28.38
	2-propylene glycol mono-1-ethyl ether	104.15	+				2.84
	01-06-1465	110.54		++			0.14
	93-06-0523	90.12					11.11
	99-04-1207	157.17			+		6.37
	96-01-0391	146.18	++	++		++	6.85
	Butynediol	86.09	++	++	++	++	0.06
	Thiodiglycol	122.19	+	+	+		4.09
	Trimethylolpropane	134.17		+	+	+	1.49
	Methanol	32.04		+		+	2.85
	Ethanol	46.07	+	+	+		137.88
	2-propanol	60.09	+				13.95
	Allyl alcohol	58.08		++	++		0.10
	2-methyl-3-butyn-2-ol	84.12			+		1.19
	Tert-butyl alcohol	74.12					1.26
Summary	Geometric Mean (Min-Max-Range)						2.78 (0.06–137.88)

+, ++ denote the presence of findings for this target; ++ strong effect = low NOEL, + less strong effect = high LOEL.

**Figure 8:**
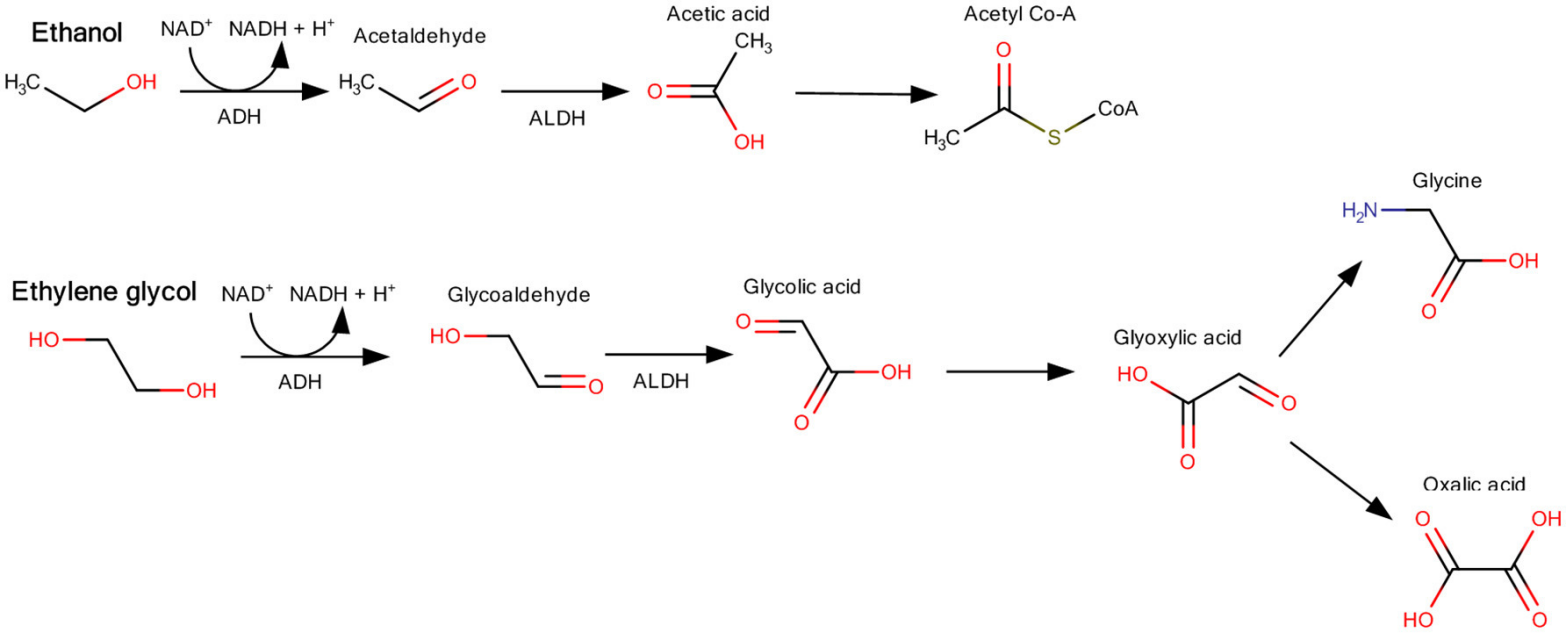
**Examples for the different metabolic pathways of alcohols (ethanol) and glycols (ethylene glycol)**. Ethylene glycol metabolism leads to glyoxylic acid and oxalic acid, which are nephrotoxic, whereas ethanol is metabolized to acetyl Co-A (Figure modified after Kraut and Kurtz, 2008; Schep et al., 2009).

Kidney toxicity of the lower molecular weight members occurs after repeated oral exposure. In the dataset of 18 members being glycols, ten showed effects on the kidney including increased kidney weight. Alcohols in the dataset did not affect the kidney with exception of weak effects of ethanol and 2-propanol. These toxicological effects are based on a specific mode of action. Ethylene glycol is metabolized by the alcohol dehydrogenase to glycol aldehyde and further to glycolic acid, which is then metabolized to glyoxylic acid and oxalic acid (Miller et al., 1984; Viinamäki et al., 2015). The kidney effect is mainly caused by the acidic metabolites (Figure 8). From the structure of the glycols, all ten members showing an effect on the kidney possess hydroxylic groups at the end of the unbranched molecules with the exception of chemicals 96-01-0391 and ethylene glycol monoethyl ether. The alcohols, most of them being also substrates for alcohol dehydrogenase, are further metabolized to smaller molecules (Figure 8), which are not excreted by the kidney and thus have no toxic effect on the kidneys. As the preselected lists of structural features do not contain a possibility to distinguish alcohols and glycols, the toxicologically distinct structures remain within the same cluster as their structural distinction cannot be made.

In Table 3, the chemicals are noted with their major targets, their molecular weight and the LOEL. Whereas, the toxicological profile is similar, the LOELs are widely spread over several orders of magnitude.

### Endpoint specific analyses

After a general descriptive cluster analysis and exemplary considerations of clusters containing certain structural features, in this part of the analysis the focus is assessing selected toxicological endpoints. A plausible clustering would reveal clusters having a toxicological fingerprint (in terms of affected endpoints) and related chemical structures. Thus, in this section we started by selected toxicological endpoints and analyzed the related structural features. As several different structures and related modes of action could cause similar effects in one and the same organ, the analysis starting from the toxicological targets may not, due to the diversity of related structures (also not frequent within the dataset), reveal meaningful clusters for some of the targets.

#### Endpoint: male reproductive organs

Male reproductive toxicity can be seen by different effects in the reproductive organs: by gross pathological parameters such as weight changes, and by histopathological parameters but also by sperm parameters (count, motility, morphology). As shown by Mangelsdorf et al. (2003), these parameters are inter-correlated but show different dose-response relationships, with organ weights and testicular histopathology being quite sensitive and indicative for male reproductive toxicants in subacute and subchronic studies. Within our dataset, overall 69 compounds were identified with more than one effect on male reproduction. These substances are spread over 41 clusters, with 18 clusters containing more than one and only six clusters containing more than two substances with several effects influencing male reproduction. The substances and clusters were further analyzed in two directions: (1) for structural features involved in toxic effects to the male reproductive organs and (2) for a correlation of effects.

In Table 4 the structural features contributing mostly to male reproductive toxicity are listed according to their incidence in substances with a reproductive toxicity profile. Here only those structures were analyzed that exhibit at least two effects in male reproductive organs. Noticeably, nitrogen moieties and to a lesser extend also carboxylic acid derivatives and amines seem to drive forward male reprotoxic effects.

**Table 4:** **Frequency of structural features in substances profiled by more than one male reproductive effect**.

Structural feature (taken from OB-FP4^*^)	**Dataset with effects on male reproduction**		**Dataset without effects on male reproduction**		***p*-value**	***p*-value adjusted**
	**Structure present**	**%**	**Structure present**	**%**		
**Nitro**	**15**	**22**	**48**	**6**	**1.6 E-007**	**1.8E-06**
Carboxylic_acid_derivative	16	23	313	38	1.5 E-002	n.s.
Amine	8	12	169	20	7.8 E-002	n.s.
Ketone	9	13	70	8	0.18	n.s.
Vinylogous_ester	9	13	159	19	0.20	n.s.
Heterocyclic	20	29	294	35	0.25	n.s.
Aromatic	39	57	514	62	0.31	n.s.
Annelated_rings	9	13	133	16	0.39	n.s.
Conjugated_double_bond	27	39	298	36	0.42	n.s.
Alcohol	7	10	99	12	0.44	n.s.
Heteroaromatic	12	17	158	19	0.47	n.s.

* SMARTs pattern for functional group classification (Open Babel; O'Boyle et al., 2011).

Additionally, the correlation of different effects was investigated by a Spearman-correlation test using log-normally distributed LOELs. Highly correlated endpoints could probably point to specific modes of action and related structures. High correlation indices were found for different effects within male reproductive organs, namely testis and epididymis: Histopathological changes in these organs were highly correlated to sperm parameter (0.86), as well as to organ weight decrease (0.95).

Analyzing the clusters for accumulation of male repro-toxic effects and suitable structural features reveals finally four clusters: 2, 6, 11, and 32. A predominant structural feature of these clusters is the nitro moiety in combination with an aromatic structure, which has well known modes of action on male reproductive organs and was already discussed with the nitro-aromatic structures above. The known modes of action include:

- Damage of Sertoli cells resulting in impaired spermatogenesis (Cave and Foster, 1990).
- Decrease of testosterone and androgen receptor expression affecting endocrine regulation of the reproductive system (Zhuang, 2008).

The correlation of histopathological, either sperm parameter or weight changes, is evident for chemicals exhibiting this kind of toxicity. Overall, 13 nitro aromatic substances were identified by the correlation analysis. This exercise shows that correlation analysis is an appropriate tool to reveal modes of action relying on several effects described in the dataset.

#### Endpoint: spleen

Effects on the spleen were seen in 23% (*n* = 208) of the compounds in 85 of 119 clusters. In 15 clusters (18%) more than 50% of the cluster members were active for the endpoint spleen. However, cluster size was limited and clusters contained maximal seven compounds (five active, two inactive). We conclude that the endpoint spleen is not covered well by the clustering algorithm. Nevertheless, it is to be noted, that some structural properties are specific for this effect (Table 5).

**Table 5:** **Structural properties for substances occurring predominantly in clusters with effects on the spleen**.

	**Dataset with effects on spleen (*n* = 208)**		**Remaining dataset (without effect on spleen, *n* = 691)**		***p*-value^a^**	***p*-value adjusted^b^**
	**Structure present**	**%**	**Structure present**	**%**		
**OB-MACCS: NA(A)A**	**125**	**60.1**	**291**	**27.8**	**5E-06**	**5.5E-05**
**OB-MACCS: N**	**140**	**67.3**	**342**	**49.5**	**6E-06**	**6.6E-05**
**OB-FP3: Ether**	**74**	**35.6**	**152**	**22**	**7.6E-05**	**8.4E-04**
OB-MACCS: Aromatic	142	68.3	411	59.5	0.022	n.s.^c^
OB-MACCS: 6M Ring	150	72.1	454	65.7	0.084	n.s.
OB-MACCS: Ring	164	78.8	509	73.7	0.131	n.s.
OB-MACCS: O > 1	122	58.7	445	64.4	0.132	n.s.
OB-MACCS: O	159	76.4	565	81.8	0.089	n.s.
OB-MACCS: X (Halogen)	64	30.8	207	30	0.823	n.s.
OB-MACCS: 6M Ring >1	55	26.4	195	28.2	0.616	n.s.
OB-MACCS: 5 M Ring	42	20.2	162	23.4	0.326	n.s.

The structural fragments origin from two lists (MACCS and FP3) as provided in Open Babel (O'Boyle et al., 2011). NA(A)A, Aliphatic N connected to any (non-hydrogen) atom, which is in turn connected to two atoms; N, Aliphatic N; Ether, Oxygen connected to two carbons. SMARTS codes and depictions of the fragments are provided in the Supplementry Material.

a X^2^-test.

b Bonferroni-Holm correction.

c n.s., not significant.

Our analysis shows that three chemical features are related to effects on the spleen. There is a statistically higher chance compared to the remaining dataset that a chemical will affect the spleen if the chemical structure contains a nitrogen (OB-MACCS:N, *p* = 0.000066) or a nitrogen followed by a branching of undefined atoms (OB-MACCS:NA(A)A, *p* = 0.000055). The third chemical feature that is significantly related to effects on the spleen is an ether moiety (OB-FP3: Ether, *p* = 0.00084). Other molecular features which were investigated are not related to effects on the spleen.

#### Endpoint: thyroid

Another endpoint of interest selected for analysis is the thyroid gland. Here the analysis is supported by considering the mode of action. In our dataset, 63 substances (7%) in 45 out of 119 clusters have effects on the thyroid gland.

**Figure 9:**
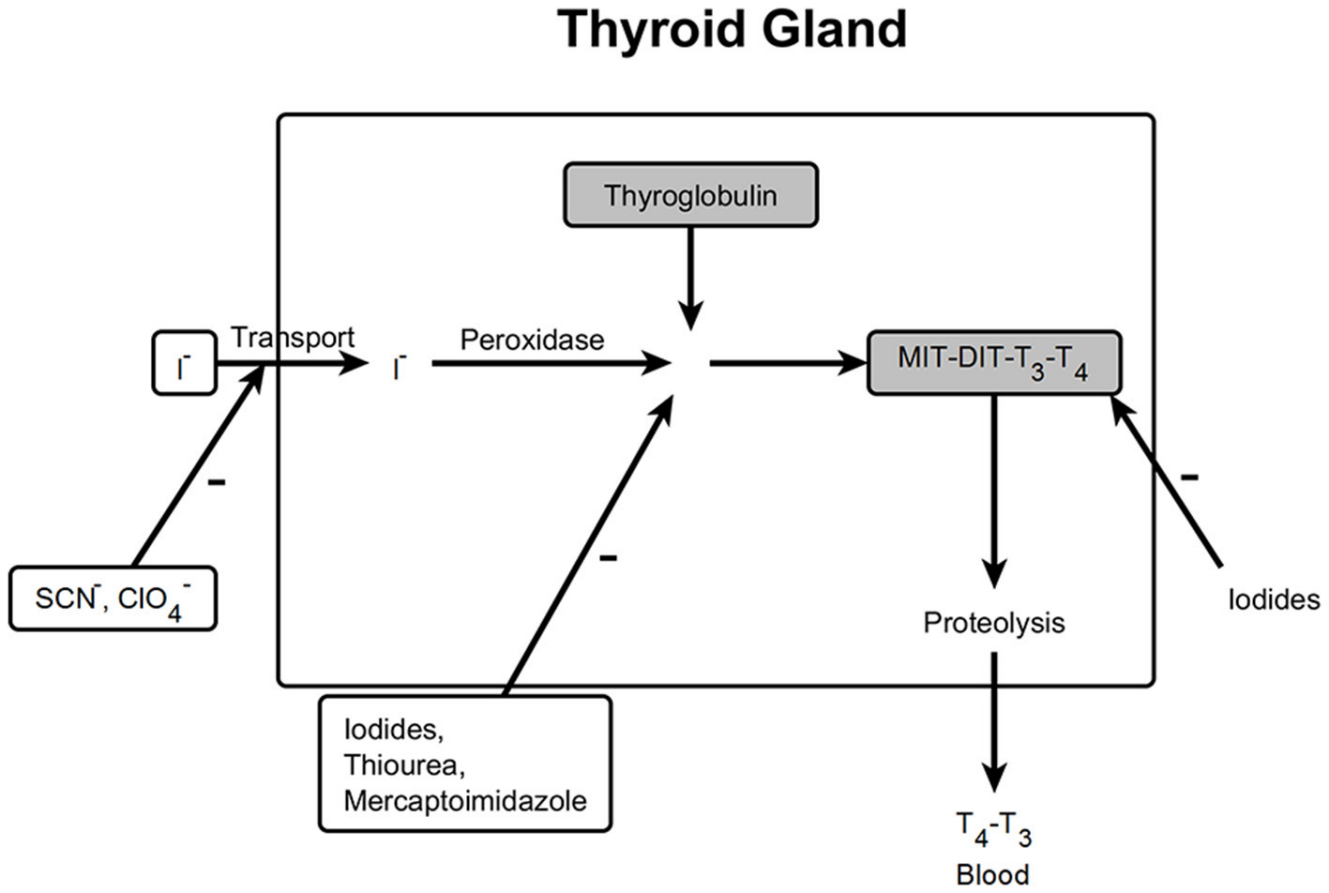
**Mechanisms which influence biosynthesis in the thyroid gland**. MIT, Monoiodotyrosine; DIT, Diiodothyrosine; T3, Triiodothyronine; T4, Tetraiodothyronine; –, inhibition.

The thyroid gland produces the hormones thyroxine and triiodothyronine, which are important regulators of basic metabolic rate. After uptake of iodine from the circulation, thyroxine is synthesized from the amino acid tyrosine and iodine involving thyroid peroxidase (Ekholm and Björkman, 1997) and triiodothyronine is produced by cleavage of one iodine from thyroxine (Figure 9).

This endpoint appeared in not more than 50% of the cluster members. It is therefore likely that the clustering algorithm does not cover the endpoint thyroid gland exactly. Hence, it was decided to analyze the 63 substances using a mechanistic approach, which is based on the biological steps and their perturbation in the production of the hormone.

In this approach, the dataset was searched for structural elements of compounds, which stimulate or inhibit thyroidal function. The organ is stimulated by low iodide concentrations whereas high concentrations block the activity. Six iodine-containing compounds were found. Two of these substances were alkyls (trifluoro-iodo-methane and 1,1,1,2,2,3,3,4,4,nona-fluoro-4-iodo-butane), in two further substances iodine was bound to a triple bond and two substances contained iodine as a substituent to a benzene ring. Only the iodine-alkyls were active. This finding might be explained by the assumption that de-iodination takes place only from those structures and iodine will be available in the systemic circulation. We assumed that iodinated benzene rings will not be de-iodinated; this behavior can be interfered from the metabolism of atrazine and its chlorinated metabolites (Brzezicki et al., 2003), where binding of chlorine to the benzene-like triazine ring is stable.

Perchlorates inhibit the iodide transport into the thyroid gland (De Groef et al., 2006). There were 14 perchlorate-like compounds in the dataset, which were all active.

Thiourea and mercaptoimidazole derivatives, which are used therapeutically, inhibit the peroxidase in the thyroid gland (Fumarola et al., 2010). Among the active structures acting on the thyroid 1 thiourea structure and 4 mercaptoimidazole derivatives were found.

Thus, for 21 out of 63 structures (41%) effects could be linked to a mechanism (iodine concentration, iodine uptake, inhibition of peroxidase). The mechanisms leading to an effect on the thyroid gland differ, depending on the different chemical structures. Hence, it is understandable that using clustering procedures based on structural and physicochemical properties, it is not possible to find a single cluster with all the members influencing the thyroid gland.

## Discussion

### Background

In the context of public awareness and regulatory demands (e.g., Cosmetics Directive, 7th Amendment to the European Union's Cosmetics Directive 76/768/EE), the need for non-animal testing is increasing. Non-animal testing methods comprise *in vitro* tests and *in silico* tools.

The OECD Guidelines for Testing of Chemicals contains regulatory guidelines with worldwide acceptance. At present, most of these tests for health effects (Section Results) are performed in animals. Only some test guidelines describe *in vitro* tests; those are restricted to tests aimed at testing local toxicity such as skin sensitization. Further accepted *in vitro* test methods are those in which genotoxicity endpoints are addressed. Until now, no *in silico* methods are validated at the OECD guideline level but the practical use of (Q)SAR approaches for regulatory purposes is supported and facilitated by the OECD (Q)SAR project. Herein, the principles for the validation of (Q)SAR models (OECD, 2007b) have been developed as well as the OECD QSAR toolbox, which is supporting grouping and read-across approaches^5^. Regulatory agencies increasingly accept *in silico* results on genotoxicity (Cassano et al., 2014; Aiba nee Kaneko et al., 2015; Jolly et al., 2015).”In the absence of toxicological data, grouping of substances and read-across approaches are encouraged in the REACH legislation to predict complex endpoints, as resulting from repeated dose toxicity testing. A data analysis performed by ECHA shows that data gaps exist for one-third (32.9%) of endpoints of the so-called phase-in substances (substances with high production volumes of 100–1000 tons per year). These data gaps have been bridged by using a read-across approach especially in the case of higher tier health effects (ECHA, 2014). It is well known that most substances with lower production volumes have not been tested in the past by repeated dose toxicity tests. Hence, it can be predicted that a percentage higher than that observed for substances with high production volumes would be without repeated toxicity testing data, e.g., substances with a production volume between 10 and 100 t/a. These observations underscore the high need for a tool to predict repeated dose toxicity data.

### Available tools

Predicting repeated dose toxicity still faces many challenges such as the lack of sufficient, good quality data and a shortage of mechanistic interpretations (Cherkasov et al., 2014). Read-across seems a promising option among other available prediction tools (Schilter et al., 2014). However, it relies to a high degree on expert knowledge (Patlewicz et al., 2013).

One of the read-across tools is the HESS system, contained in the OECD QSAR Toolbox. With this tool grouping and read-across for about 40 categories and 20 structural alerts can be performed for repeated toxicity endpoints in mammalians^6^ (Yamada et al., 2012, 2013a,b, 2014; Sakuratani et al., 2013a,b). The further evaluation of the predictions using the HESS system remains an open task.

Another framework that aids identifying analogs and rating their suitability for read-across is presented by Wu et al. (2010). The prediction framework Lazar (Lazy Structure-Activity Relationships) resembles an automated read-across procedure (Maunz and Helma, 2008; Maunz et al., 2013). It predicts a query compound by performing a similarity search on the training dataset and building a local (Q)SAR model using only similar compounds. Low et al. (2013) present a chemical-biological read-across (CBRA) approach that infers toxicity based on structural similarity but also on biological similarity. Similar to Lazar, the method uses instance-based local models with similarity warning and present neighbors. However, this method is not a pure *in silico* method, as biological responses of compounds have to be measured *in vitro* in short-term assays.

Furthermore, there are commercially available tools, for example DEREK (Lhasa Limited, UK) and TOPKAT (Accelrys Inc., San Diego, USA; Venkatapathy et al., 2004; Rupp et al., 2010) which may be used for read-across purposes. Whereas, DEREK provides qualitative information on the target organ toxicity based on similar patterns or structural alerts, TOPKAT is performing predictions on the basis of structural similarity and claims to be quantitative; it predicts LOAELs. Testing the TOPKAT tool by using the same ELINCS dataset as in the current analysis, revealed dissatisfying results showing that the use of a refined prediction tool is highly warranted (Rupp et al., 2010).

### Our approach/project

There is an obvious need for prediction tools. However, all the available tools have limitations and there seems to be room for improvement in read-across tools. In the project presented in this paper, a clustering algorithm for repeated dose toxicity was developed in a bi-dimensional approach, combining physical structural properties and organ toxicity data, an idea, which has been also discussed in the paper by Maunz et al. (2013). This was achieved by applying PCTs, a decision-tree based clustering tool that can handle multiple endpoints simultaneously. Applying this tool yielded a hierarchical clustering of our dataset, including a description of the structural properties that were used to assign compounds into sub-clusters of compounds with similar rat repeated dose toxicity profiles. To characterize the compounds we selected lists of structural features and two simple PC-descriptors (molecular weight and log P) as features for constructing the PCT model with homogenous toxicological fingerprints as criterion of the clustering process. The features, which are employed to create nodes within the tree to divide compounds into sub-clusters, yield a transparent description for each cluster.

It is a distinguishing feature of this project that the toxicological dataset used for the clustering algorithm is a highly refined and curated dataset of high quality. In addition, the number of chemicals is higher than in any other curated dataset, containing data from repeated dose toxicity testing performed according to OECD guidelines. To prepare the common dataset, which was derived from two sources, a common glossary has been developed and the data have been curated to allow using the whole dataset of 899 substances. In this dataset, LOELs from 460 different organ-effect-combinations were available. This information was finally condensed to 28 endpoints describing organ-effect combinations. The dose-response relationship, namely the LOELs, was discretized per endpoint resulting into substances with high and low potency. Missing values were filled by imputation, predicting the missing values with a dedicated prediction model.

Within the curation steps, several shortcomings of the data base which would have negatively influenced the clustering results had to be dealt with. The noise of underrepresented and/or unspecific endpoints was reduced by restriction to 28 aggregated organ-effect combinations. It is well-known that dose spacing has major impact on the LOEL values causing imbalances in the resulting LOELs of different substances. We overcame this problem by the process of discretizing the LOELs per endpoint. We applied imputation to limit the impact of missing values on the clustering outcome.

As it is shown in this publication, the final results were achieved by several rounds of iterations and optimizations.

### Lessons learned

From our study, the simplest predictors for a high probability of low toxicity are a molecular weight above 500 and/or a log P below 0 or above 5. It is interesting to note that in a retrospective study on their opinions, the SCCS found a similar molecular weight (above 500 Da) and similar log P (between below −1 and above 4) for a low or even very low dermal absorption and hence toxicity (SCCS, 2015). Further support is provided by several authors for the importance of log P for toxicity resulting in screening drug candidates (Hughes et al., 2008; Greene et al., 2010; Lu et al., 2012). Physicochemical properties are important parameters to predict diffusion processes through biological membranes and therefore also relevant for the prediction of chemical uptake. In this context, our findings demonstrate the role of internal exposure for toxicity. In order to include the absorption properties in our cluster information, it is relevant to implement physicochemical descriptors into the clustering process.

The clusters derived in this clustering approach have been analyzed from a toxicological point of view. This evaluation showed in the 28 clusters with a structural similarity of 100%, that in 60% of these, there was a toxicological similarity of ≥75% (Supplementary Table 1). However, considering that the clustering procedure resulted in 119 clusters for which only 28 (14%) gave a consistent structural and toxicological similarity; there is room for improvement in the decision hierarchy.

Several factors increase the complexity in the relationship between structural and toxicological similarity, leading to the consequence that in only 60% of clusters with 100% structural similarity, there was also a similarity in the toxicological profile:

- First, as has been shown for example with the glycols, metabolism plays an important role for the toxicity. However, reliable tools to predict metabolic pathways, which would give useful information, are not yet sufficiently developed to be fit for purpose (Anger et al., 2012). The importance and the difficulties in prediction of metabolism have also been shown for the hepatotoxicity of allyl esters (Yamada et al., 2013b).
- Secondly, as demonstrated by the example of the effects on the thyroid, there are several mechanisms, which may show the same effect. Hence, several structural properties, each related to a separate mode of action or AOP may result in the same effect at the organ level (e.g., effects on the thyroid weight; Yamada et al., 2013a).

From our analysis we learned about the additional value of implementing the toxicity into the clustering procedure by the example of compounds with nitro moiety. This example shows that a better partitioning in clusters can be reached if toxicity targets, potency, and mode of action have been implemented into the clustering procedure.

### Categories suitable for read-across

We have identified some clusters with a high degree of structural similarity (assessed as 100%) as also showing a high degree of toxicological similarity. These clusters could be taken into consideration as new categories in a qualitative read-across (Supplementary Table 1). The structural and toxicological similarities give a reasonable basis for further evaluation of the linking mode of action and the complete toxicological fingerprints taking into account the role of metabolism. Furthermore, clusters in which the ratio between the geometric mean of LOEL and the lower end of the LOEL range do not exceed 5 could be used in a quantitative read-across approach (Table 1). If the geometric mean of the cluster LOEL is taken to predict the LOEL of a chemical substance falling in one of the indicated clusters, the true LOEL might be higher or lower than the (geometric) mean. From a regulatory point of view, it would only matter, if the true LOEL were lower than predicted. The use of an additional uncertainty factor might be necessary to be on the safe side. As a value of 5 was chosen as cut-off criterion for the ratio in selecting the clusters for a quantitative read across, using an additional factor of 5 would be sufficient to take into account this uncertainty.

### Conclusion

In conclusion, a new method for clustering databases on chemical substances with accompanying toxicological information according to their chemical properties was introduced, by developing a decision tree that uses structural and toxicological information to calculate the similarity of the test compound. The procedure is intended to support a read-across approach. In analyzing the clustering results from the toxicological point of view, we did find out that in some of the clusters, the cluster members have common mechanisms of action and that the toxicological target and effects are similar. In these cases, the clustering leads not only to structurally but also to toxicologically meaningful results. We propose that these clusters are suited for a read-across. These clusters encompass clusters of category 1 (100% structural similarity/100% toxicological similarity) and category 2 (100% structural similarity/≥75% toxicological similarity). For others, the clustering resulted in disparate toxicity and/or chemical structures of the cluster members without a common mechanism of action. Furthermore, some clusters were identified in which the ratio between the geometric mean of the cluster LOEL and the LOEL of the lower end of the range was small (≤ 5). We propose that those clusters might be used for a quantitative read-across. In risk assessment, the additional uncertainty of this approach can be taken into consideration by applying an additional uncertainty factor of 5.

This publication shows that structural analyses combined with effect analyses give the advantage of identifying mechanism related to structures and hence provide a tool for improving the prediction of relevant structures. However, it is clear that further improvement is needed, e.g., by incorporating data on metabolism and bioavailability. For further development it could also be envisaged to add additional toxicological data such as environmental concentrations or *in-vitro* data if available thus enabling comparison between LOELs and other data for special applications. The insights provided by this project will help research in this field by eliciting the requirements for even more advanced tools.

## Availability

The model is freely available^7^ and can be applied to assign untested compounds to clusters. But there is one restriction: the confidential data including the chemical structures of the ELINCS database are not shown because of confidentially reasons.

## Author contributions

MB, MG, FP, UG, SK, and AB developed the concept; MB and FP curated the dataset; MG, SK, CH, AM, and MS were involved in several steps of the clustering procedure; MB, MG, UG, and AB evaluated the clustering results from toxicological point of view; MB and SK developed the discretization and handling of missing value procedures; all authors contributed to the manuscript which was finalized by MB, MG, FP, UG, SK, and AB.

### Conflict of interest statement

The authors declare that the research was conducted in the absence of any commercial or financial relationships that could be construed as a potential conflict of interest.

## Footnotes

^1^In this paper we use the expression generic target organ to indicate that there is the target organ known (i.e., liver), however no specific effect (e.g., liver weight increase) was observed. The expression “specific target-organ effect” is used when a specific effect is described (e.g., liver necrosis). MOA means mode of action which indicates a description of the route by which the adverse effect is mediated, whereby however several intermediate steps are not known. The expression fingerprint is used to indicate a chemical or chemical group specific toxicological pattern with toxicological effects in several organs/tissues.

^2^accessible at http://mlc-reach.informatik.uni-mainz.de

^3^http://www.fraunhofer-repdose.de

^4^http://mlc-reach.informatik.uni-mainz.de

^5^http://www.oecd.org/chemicalsafety/risk-assessment/theoecdqsartoolbox.htm query from 18^th^ of August 2016

^6^http://www.nite.go.jp/en/chem/qsar/hess-e.html

^7^at http://mlc-reach.informatik.uni-mainz.de
