# Supplementary material for: Innovative Strategies to Develop Chemical Categories Using a Combination of Structural and Toxicological Properties
Source: Front Pharmacol. 2016 Sep 21;7:321. doi: 10.3389/fphar.2016.00321 (PMC5030828; doi:10.3389/fphar.2016.00321)
Supplement: Supplementary file 1 [file DataSheet1.DOCX]

Supplementary Material

Innovative Strategies to Develop Chemical Categories Using a Combination of Structural and Toxicological Properties

**Batke M.^1°^, Gütlein M.^2°^, Partosch F.^3°^, Gundert-Remy U.^4*^, Helma C. ^5^, Kramer S.^2^, Maunz A.^6^, Seeland M.^7^, Bitsch A.^1^**

^1^Fraunhofer Institut für Toxikologie und Experimentelle Medizin (Hannover, Germany), ^2^Universität Mainz (Mainz, Germany), ^3^Institut für Arbeits-, Sozial- und Umweltmedizin, Universitätsmedizin Göttingen (Göttingen, Germany), ^4^Institut für Klinische Pharmakologie und Toxikologie, Charité Universitätsmedizin Berlin (Berlin, Germany), ^5^In silico toxicology GmbH (Basel, Switzerland), ^6^Oncotest GmbH (Freiburg, Germany) ^7^Technische Universität München (München, Germany)

°These authors contributed equally to this paper

*Corresponding author: Prof. Dr. U. Gundert-Remy

e-mail: [Ursula.Gundert-Remy@charite.de](mailto:Ursula.Gundert-Remy@charite.de)

**Supplementary Table 1** Clusters with a structural similarity of 100 %. For each cluster we give the number of chemicals, the common structural feature, common target endpoints with the degree of toxicological similarity, the geometric mean of LOEL (mmol) and its range.

| Category | Number of Cluster | Number of Chemicals | Structural Feature | Structural Similarity [%] | Common Target | Toxicological Similarity [%] | LOEL [mmol] (geometric mean; range) |
| --- | --- | --- | --- | --- | --- | --- | --- |
| 1 | 13 | 6 | methylphenols | 100 | CNS | 100 | 0.58 (0.32-1.39) |
| 1 | 15 | 3 | phosporic acid derivatives, aromatic | 100 | RBC | 100 | 0.04 (0.001-1) |
| 1 | 82 | 3 | alkylchloride | 100 | CNS | 100 | 0.69 (0.09-3.87) |
| 1 | 86 | 4 | urethane | 100 | liver | 100 | 1.2 (0.15-2.34) |
| 1 | 87 | 3 | nitrophenols und -anilins | 100 | liver | 100 | 0.21 (0.05-0.66) |
| 1 | 102 | 5 | amines, anilines | 100 | liver, liver weight, kidney weight | 100 | 0.14 (0.01-5.54) |
| 1 | 107 | 3 | alcoholes | 100 | kidney weight, liver weight | 100 | 0.03 (0.01-0.09) |
| 1 | 115 | 3 | aminophenols | 100 | bw, female repro, kidney, kidney weight, liver, liver weight | 100 | 0.47 (0.04-6.48) |
| 2 | 8 | 6 | acrylic acid derivatives | 100 | liver | 83 | 0.33 (0.02-4.65) |
| 2 | 75 | 6 | glycolethers | 100 | liver | 83 | 0.75 (0.14-2.84) |
| 2 | 54 | 5 | amines | 100 | kidney | 80 | 0.92 (0.08-3.88) |
| 2 | 78 | 10 | aromatic esters | 100 | liver | 80 | 0.4 (0.04-2.95) |
| 2 | 117 | 5 | alcoholes | 100 | kidney, liver | 80 | 0.7 (0.06-3.27) |
| 2 | 16 | 4 | aromatic phenol derivatives without other substituents | 100 | liver | 75 | 0.87 (0.23-13.50) |
| 2 | 44 | 4 | carboxylesther | 100 | liver | 75 | 1 (0.22-3.85) |
| 2 | 56 | 4 | nitroaromates, OC(N)C | 100 | liver | 75 | 0.48 (0.06-4.02) |
| 2 | 99 | 4 | chlorinated aromates | 100 | liver (low toxicity) | 75 | 0.05 (0.004-0.32) |
| 3 | 5 | 6 | chloro acetic acid and chloroformic acid derivatives | 100 | liver | 67 | 0.26 (0.01-4.72) |
| 3 | 9 | 6 | ether, aliphatic | 100 | liver | 67 | 20. 9 (2.82-185.91) |
| 3 | 53 | 3 | nitroaromates | 100 | CNS | 67 | 0.77 (0.24-2.01) |
| 3 | 61 | 3 | azoles | 100 | kidney | 67 | 0.52 (0.49-0.59) |
| 3 | 70 | 3 | heterocyclic carbonic acid | 100 | spleen | 67 | 0.03 (0.005-0.2) |
| 3 | 32 | 8 | nitrotoluenes (toxic) or dimethylnitrobenzenes (less toxic) | 100 | RBC | 63 | 0.23 (0.03-0.99) |
| 3 | 10 | 7 | small aliphatic aldehydes | 100 | kidney | 57 | 1.54 (0.03-11.81) |
| 3 | 57 | 10 | ether | 100 | liver | 50 | 0.16 (0.00008-5.11) |
| 3 | 110 | 4 | halogenated benzoic acid derivatives | 100 | low toxicity | 50 | 0.45 (0.06-2.04) |
| 3 | 37 | 5 | xanthene | 100 | n.d. | 40 | 0.14 (0.02-0.68) |
| 3 | 89 | 8 | chloro alkanes, part. chlorinated twice and/or with double bond | 100 | n.d. | 20 | 1.52 (0.14-23.24) |
