# Supplementary material for: Innovative Strategies to Develop Chemical Categories Using a Combination of Structural and Toxicological Properties
Source: Front Pharmacol. 2016 Sep 21;7:321. doi: 10.3389/fphar.2016.00321 (PMC5030828; doi:10.3389/fphar.2016.00321)

Supplementary Material

Innovative Strategies to Develop Chemical Categories Using a Combination of Structural and Toxicological Properties

**Batke M.^1°^, Gütlein M.^2°^, Partosch F.^3°^, Gundert-Remy U.^4*^, Helma C. ^5^, Kramer S.^2^, Maunz A.^6^, Seeland M.^7^, Bitsch A.^1^**

^1^Fraunhofer Institut für Toxikologie und Experimentelle Medizin (Hannover, Germany), ^2^Universität Mainz (Mainz, Germany), ^3^Institut für Arbeits-, Sozial- und Umweltmedizin, Universitätsmedizin Göttingen (Göttingen, Germany), ^4^Institut für Klinische Pharmakologie und Toxikologie, Charité Universitätsmedizin Berlin (Berlin, Germany), ^5^In silico toxicology GmbH (Basel, Switzerland), ^6^Oncotest GmbH (Freiburg, Germany) ^7^Technische Universität München (München, Germany)

°These authors contributed equally to this paper

*Corresponding author: Prof. Dr. U. Gundert-Remy

e-mail: [Ursula.Gundert-Remy@charite.de](mailto:Ursula.Gundert-Remy@charite.de)

## Depiction of structural fragments

**Fragment 1 — OB-FP4:Aromatic**

SMARTS: a

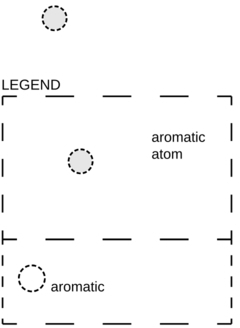


**Fragment 2 — OB-FP4:Conjugated_double_bond**

SMARTS: *=*[*]=,#,:[*]

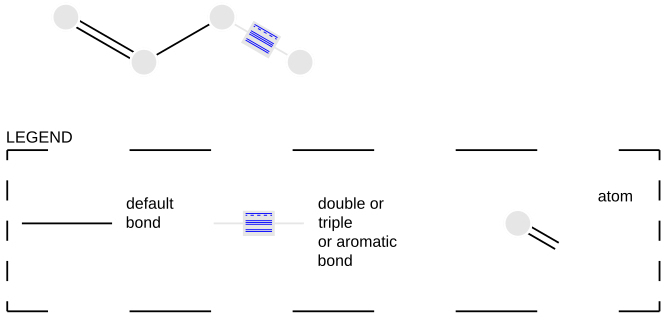


**Fragment 3 — OB-FP4:Primary_carbon**

SMARTS: [CX4H3][#6]

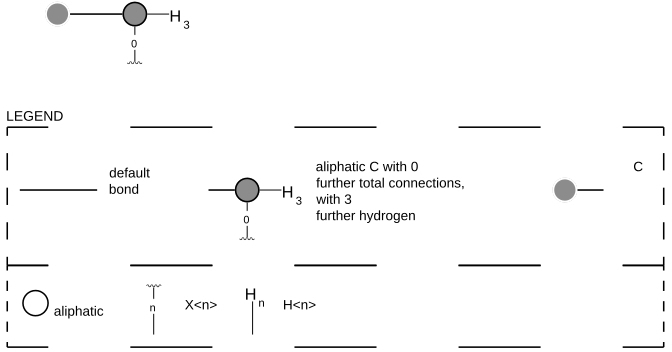


**Fragment 4 — OB-FP4:Secondary_carbon**

SMARTS: [CX4H2]([#6])[#6]

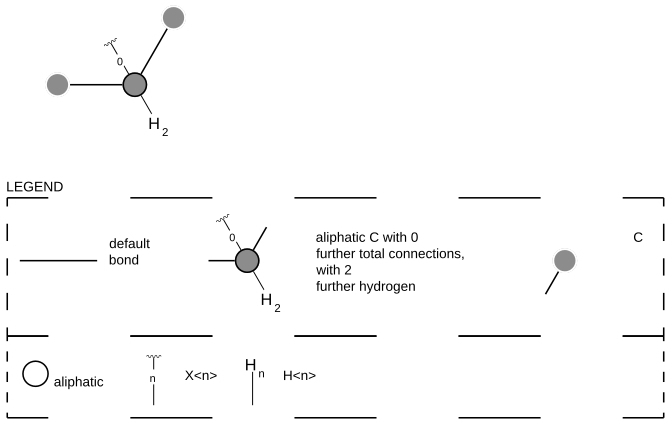


**Fragment 5 — OB-FP4:Heterocyclic**

SMARTS: [!#6;!R0]

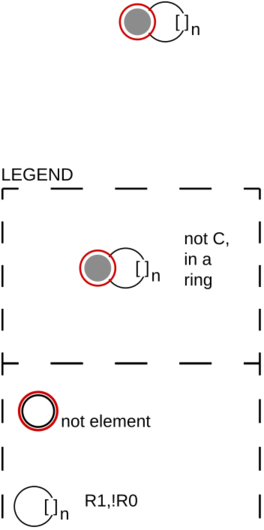


**Fragment 6 — OB-FP4:Nitro**

SMARTS: [$([NX3](=O)=O),$([NX3+](=O)[O-])][!#8]

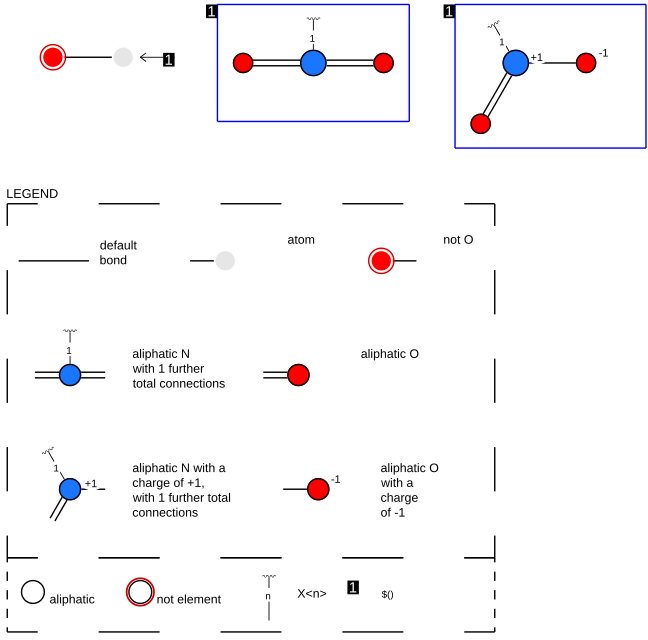


**Fragment 7 — OB-FP4:Carboxylic_acid_derivative**

SMARTS: [$([#6X3H0][#6]),$([#6X3H])](=[!#6])[!#6]

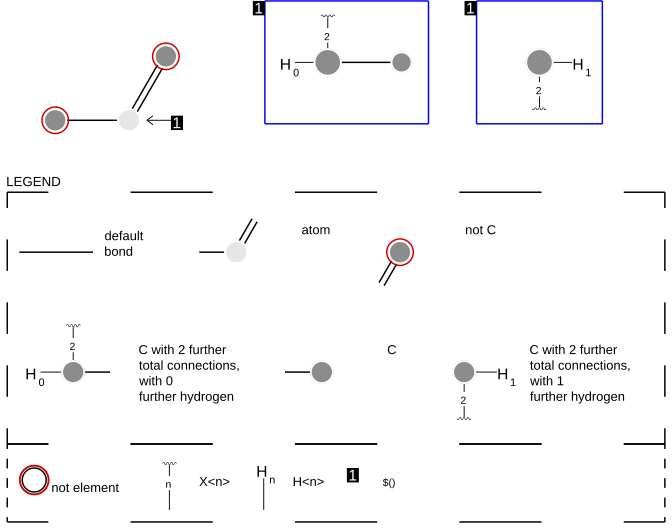


**Fragment 8 — OB-FP4:CH-acidic**

SMARTS: [$([CX4;!$([H0]);!$(C[!#6;!$([P,S]=O);!$(N(~O)~O)])][$([CX3]=[O,N,S]),$(C#[N]),$([S,P]=[OX1]),$([NX3]=O),$([NX3+](=O)[O-]);!$(*[S,O,N;H1,H2]);!$([*+0][S,O;X1-])]),$([CX4;!$([H0])]1[CX3]=[CX3][CX3]=[CX3]1)]

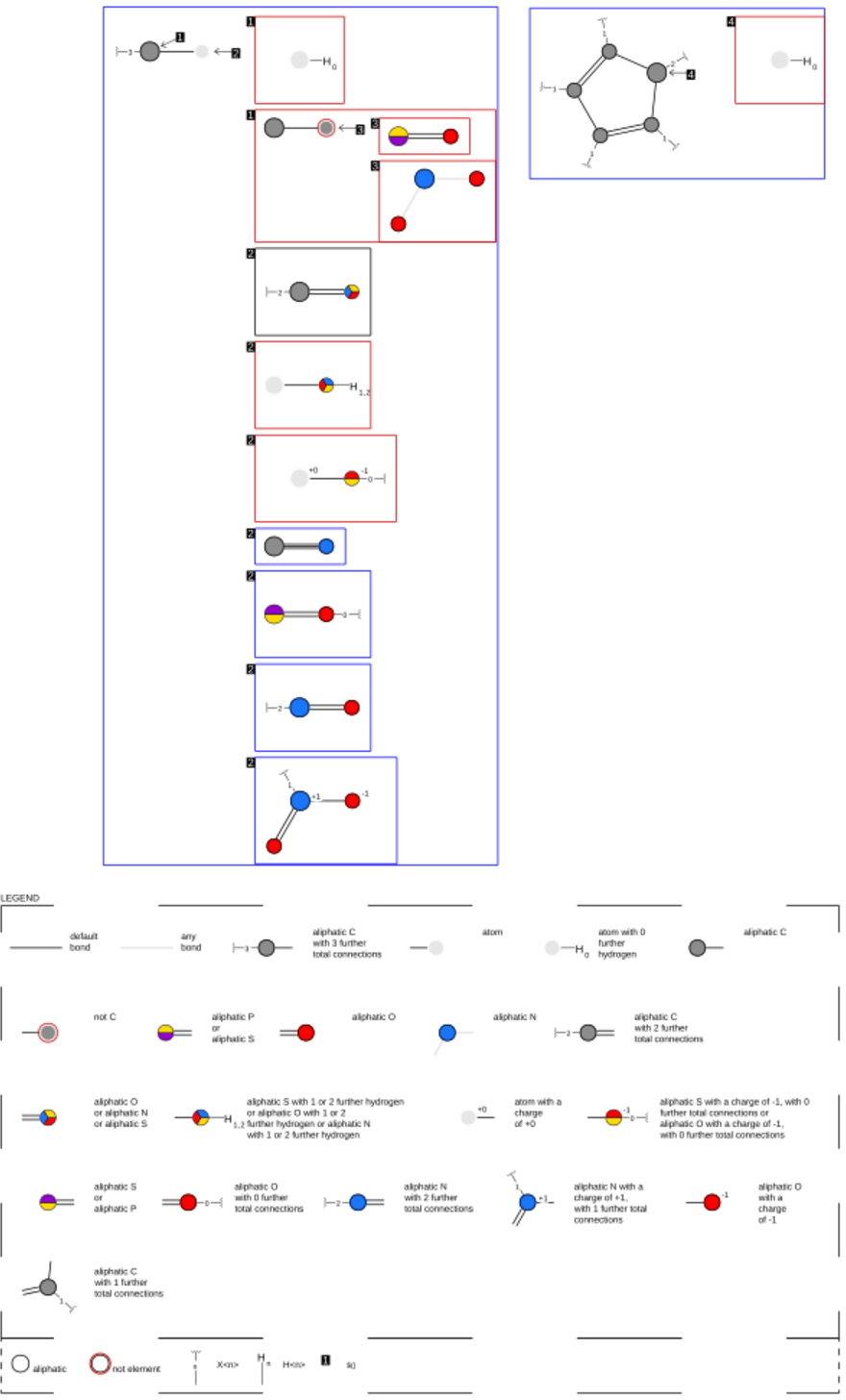


**Fragment 9 — OB-FP4:Heteroaromatic**

SMARTS: [a,!c]

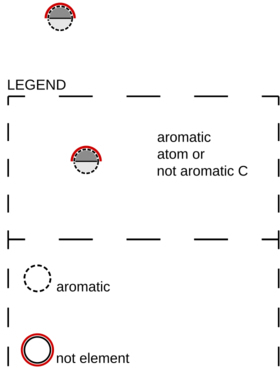


**Fragment 10 — OB-FP4:Vinylogous_ester**

SMARTS: [#6X3](=[OX1])[#6X3]=,:[#6X3][#6;!$(C=[O,N,S])]

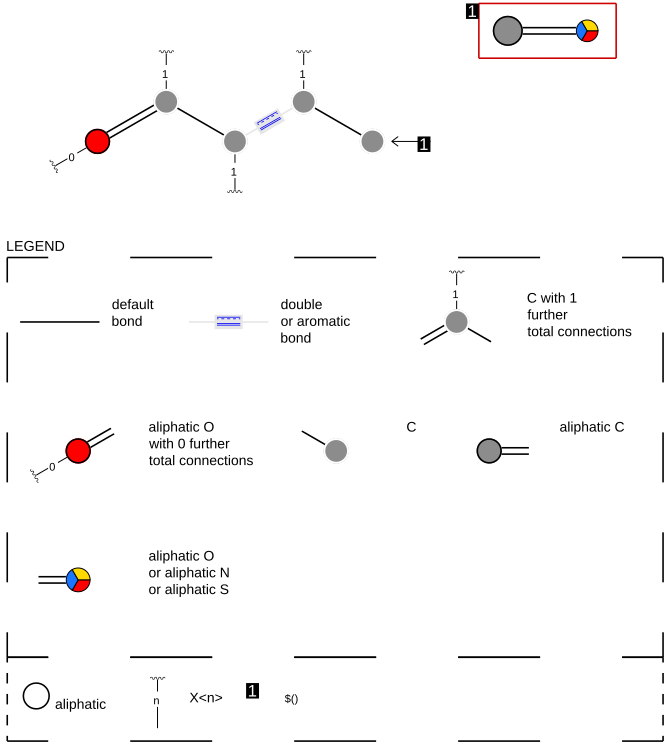


**Fragment 11 — OB-FP4:Ketone**

SMARTS: [#6][CX3](=[OX1])[#6]

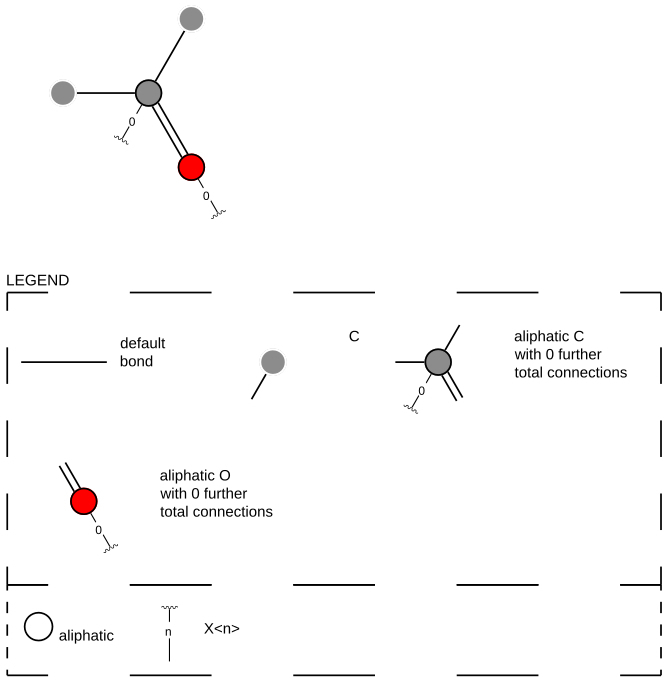


**Fragment 12 — OB-FP4:Amine**

SMARTS: [NX3+0,NX4+;!$([N]~[!#6]);!$([N]*~[#7,#8,#15,#16])] 

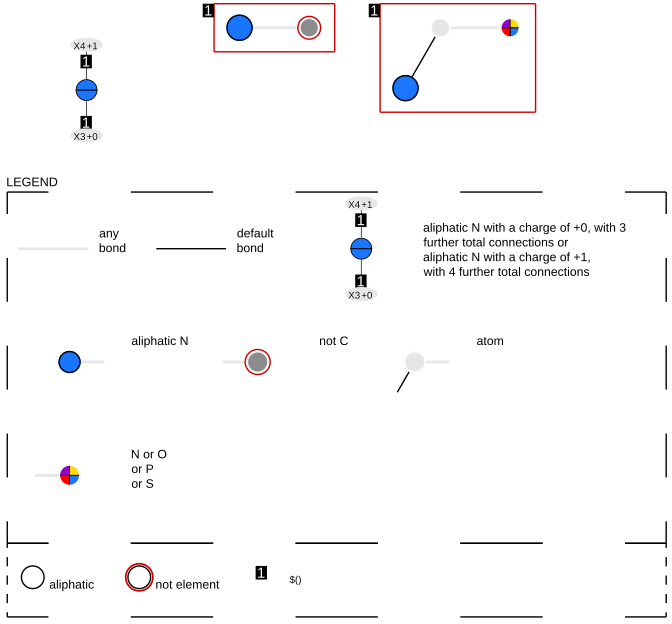


**Fragment 13 — OB-FP4:Alkene**

SMARTS: [CX3;$([H2]),$([H1][#6]),$(C([#6])[#6])]=[CX3;$([H2]),$([H1][#6]),$(C([#6])[#6])]

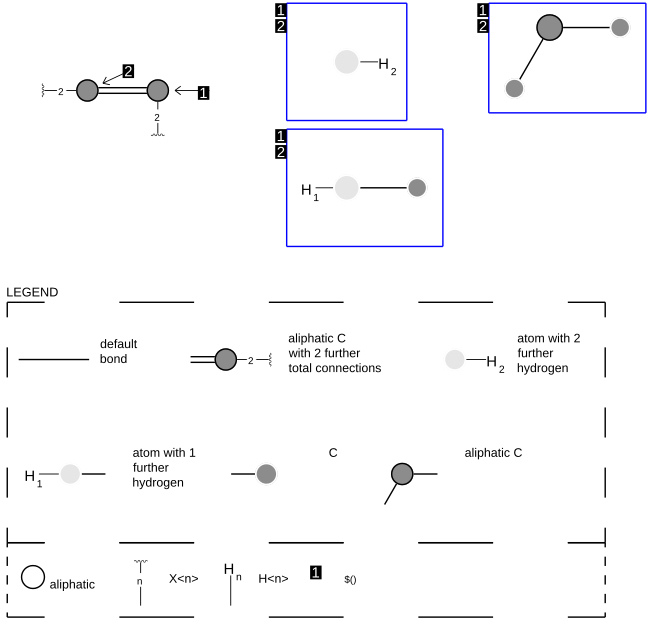


**Fragment 14 — OB-MACCS:NA(A)A**

SMARTS: [#7]~*~*~*~[#7]

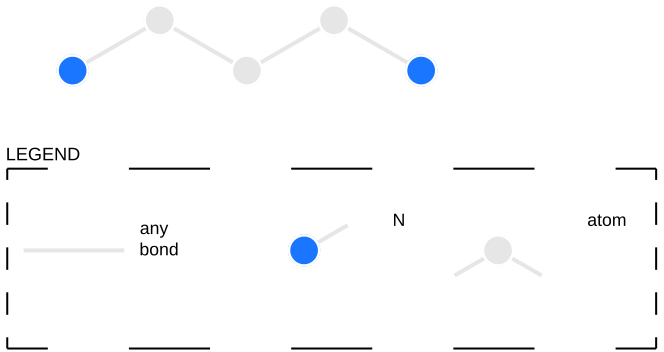


**Fragment 15 — OB-MACCS:N**

SMARTS: [#7]

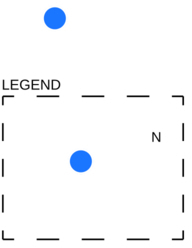


**Fragment 16 — OB-MACCS:Aromatic**

SMARTS: a

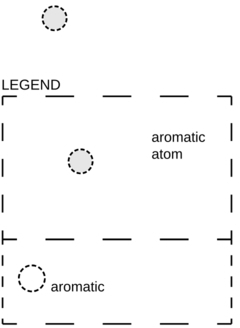


**Fragment 17 — OB-MACCS:6M Ring**

SMARTS: *1~*~*~*~*~*~1

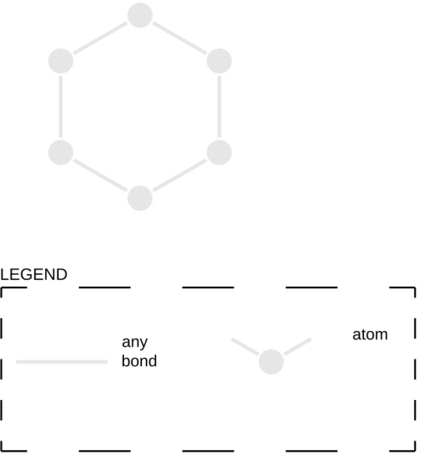


**Fragment 18 — OB-MACCS:Ring**

SMARTS: [R]

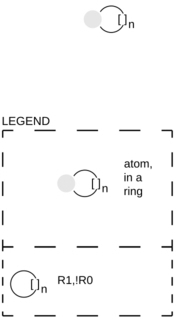


**Fragment 19 — OB-MACCS:O > 1**

SMARTS: [#8]

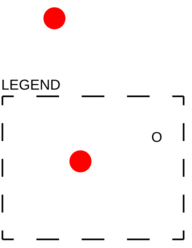


**Fragment 20 — OB-MACCS:O**

SMARTS: [#8]

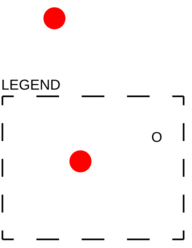


**Fragment 21 — OB-MACCS:X (Halogen)**

SMARTS: [F,Cl,Br,I]

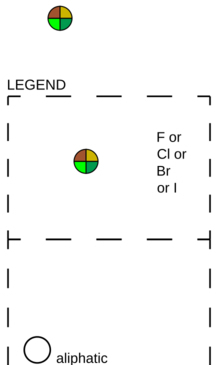


**Fragment 22 — OB-MACCS:6M Ring > 1**

SMARTS: *1~*~*~*~*~*~1

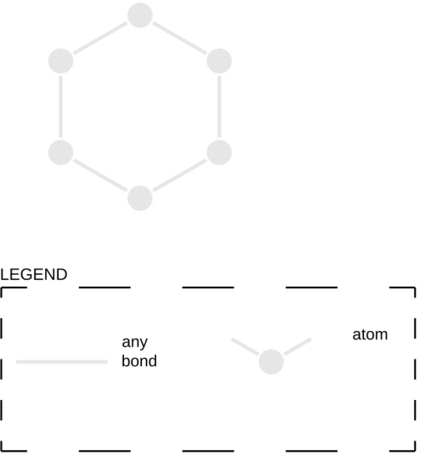


**Fragment 23 — OB-MACCS:5 M Ring**

SMARTS: *1~*~*~*~*~1

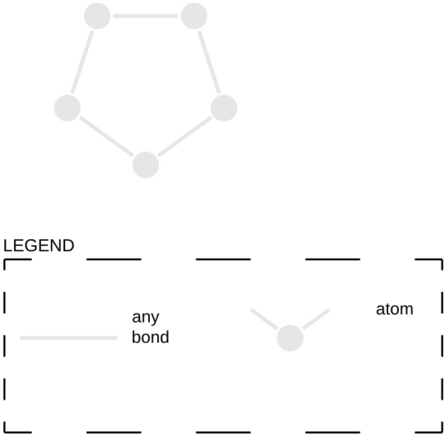


**Fragment 24 — OB-FP3:Ether**

SMARTS: [#6]O[#6]

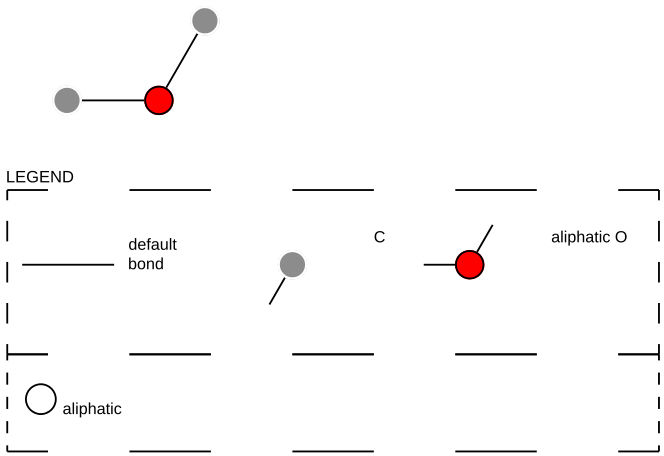

Supplement: Supplementary file 2 [file DataSheet2.DOCX]
