# Supplementary material for: Innovative Strategies to Develop Chemical Categories Using a Combination of Structural and Toxicological Properties
Source: Front Pharmacol. 2016 Sep 21;7:321. doi: 10.3389/fphar.2016.00321 (PMC5030828; doi:10.3389/fphar.2016.00321)
Supplement: Supplementary file 3 [file DataSheet3.DOCX]

Supplementary Material

Innovative Strategies to Develop Chemical Categories Using a Combination of Structural and Toxicological Properties

**Batke M.^1°^, Gütlein M.^2°^, Partosch F.^3°^, Gundert-Remy U.^4*^, Helma C. ^5^, Kramer S.^2^, Maunz A.^6^, Seeland M.^7^, Bitsch A.^1^**

^1^Fraunhofer Institut für Toxikologie und Experimentelle Medizin (Hannover, Germany), ^2^Universität Mainz (Mainz, Germany), ^3^Institut für Arbeits-, Sozial- und Umweltmedizin, Universitätsmedizin Göttingen (Göttingen, Germany), ^4^Institut für Klinische Pharmakologie und Toxikologie, Charité Universitätsmedizin Berlin (Berlin, Germany), ^5^In silico toxicology GmbH (Basel, Switzerland), ^6^Oncotest GmbH (Freiburg, Germany) ^7^Technische Universität München (München, Germany)

°These authors contributed equally to this paper

*Corresponding author: Prof. Dr. U. Gundert-Remy

e-mail: [Ursula.Gundert-Remy@charite.de](mailto:Ursula.Gundert-Remy@charite.de)

## Discretization method

The following pseudo code describes a clustering-based method to determine a threshold for binary discretization. The method detects a threshold according to the provided data distribution within a pre-defined range of to 1.5-2.0 μmol. Compounds with a LOEL lower or equal to this threshold are categorized as high-potency compounds; compounds above this threshold are categorized as low-potency compounds. An arbitrary cluster algorithm (with configurable number of clusters) could be employed, we have applied *k*-Means clustering with random restarts.

E_sub-accute_ ← list of numeric endpoint values from sub-accute studies
E_2×sub-chronic_← {x|y ∈ E_sub-chronic_ ∧ x = 2y}
E ← E_sub-accute_ ∪ E_2×sub-chronic_n ← 2

**while** no threshold t found **do** C ← cluster(E,n) // *returns list of clusters, sorted according to endpoint value* // now, *determine if cluster boundary within range exists*
 **for** i in 1.. n-1 **do**
 **if** max _E_(c_i_) ≥ 1.5µ-mol **and** min _E_(c_i+1_) ≤ 2.0µ-mol **then** t ← (max _E_(c_i+1_) + min _E_(c_i_)) / 2
 **end if
 end for** n ← n +1
**end while**

t_sub-accute_ ← t
t_sub-chronic_← t/2
